# Cross-Sectional Analysis of Infant Diet, Outcomes, Consumer Behavior and Parental Perspectives to Optimize Infant Feeding in Response to the 2022 U.S. Infant Formula Shortage

**DOI:** 10.3390/nu16050748

**Published:** 2024-03-05

**Authors:** Karla Damian-Medina, Karina Cernioglo, Maha Waheed, Dina M. DiMaggio, Anthony F. Porto, Jennifer T. Smilowitz

**Affiliations:** 1 Department of Nutrition, University of California Davis, Davis, CA 95616, USA; kadamianmedina@ucdavis.edu (K.D.-M.); kkurudimov@ucdavis.edu (K.C.); mbwaheed@ucdavis.edu (M.W.); 2 NY Langone Medical Center, New York, NY 10016, USA; dina.dimaggio@nyulangone.org; 3 School of Medicine, Yale University, New Haven, CT 06510, USA; anthony.porto@yale.edu

**Keywords:** breastfeeding, consumer behavior, equity, feeding practices, food security, health, government and regulatory policies, infant formula shortage, lactation, pediatrics

## Abstract

In May of 2022, millions of U.S. parents encountered uncertainty in safely feeding their infants due to the infant formula shortage. Methods: An anonymous, electronic, cross-sectional, retrospective survey was used. Results: U.S. parents (*n* = 178) whose infants were ~10 weeks old during the shortage completed the survey. Of parents, 81% switched formulas during the shortage, 87% switched because they could not find the formula they typically used, 34% switched 3–5 times, 29% of parents visited ≥4 stores/24 h and 26% of parents traveled >20 miles/24 h to purchase formula. Use of infant formula increased (*p* < 0.01); in infants requiring specialty formula, use of intact cow’s milk formula increased (*p* < 0.05) and use of premature infant formulas decreased (*p* < 0.05). Infants relying on specialty formulas experienced at least one undesirable outcome compared with non-specialty users. Parents used social media, relatives/friends and healthcare providers for support during the shortage, but their helpfulness scores were suboptimal. Parents reported the need for greater infant formula availability, free prenatal lactation education and postpartum lactation support. Conclusions: Government, regulatory and healthcare policy oversight are needed to protect the infant feeding system, including more commercially available products, access to banked donor milk and lactation support.

## 1. Introduction

In 2022, the United States (U.S.) encountered a severe shortage of infant formula with a recall initiated by Abbott Nutrition, one of the largest U.S. infant formula manufacturers that supplies 40% of the nation’s infant formula [1]. Abbott recalled multiple brands of its powdered formula products due to bacterial contamination from *Cronobacter sakazakii* [2]. Additionally, Abbott also voluntarily closed one of the country’s largest manufacturing facilities in Michigan, which was linked to the contamination [3]. These events were exacerbated by supply chain disruptions related to the COVID-19 pandemic, along with restrictive trade and tariff policies in the U.S. that led to a reduction of available infant formula [4]. By the end of May 2022, the shortage reached its height, with a national out-of-stock rate for infant formula as high as 90% in several states [5] which left millions of parents to face uncertainty in safely feeding their infants [6,7]. In response, the U.S. government introduced “Operation Fly” to assist families in accessing safe imported infant formulas. Under Operation Fly, the U.S. Department of Agriculture (USDA), the Department of Health and Human Services (HHS) and the General Services Administration (GSA) partnered with other nations that meet U.S. health and safety standards to import infant formula to the U.S. 

The 2022 infant formula shortage was particularly challenging for vulnerable populations such as families from low-income communities that participate in the Special Supplemental Nutrition Program for Women, Infants, and Children (WIC) [1] that heavily depend on infant formula (consuming >50% of U.S.-produced formula) [8,9] and infants in need of specialty formulas due to medical or other conditions [10]. Infants that require specialty formulas due to metabolic or medical conditions such as inborn error of metabolism, low birth weight or other medical or dietary conditions represent approximately 6% of infants that use infant formula in the U.S. [11].

Due to the inaccessibility of infant formula, healthcare providers expressed concerns that parents would resort to unsafe infant feeding practices, contrary to recommendations of the American Academy of Pediatrics (AAP), Centers for Disease Control and Prevention (CDC) and Food and Drug Administration (FDA). Such unsafe infant feeding practices include: (1) diluting formula with water [12]; (2) preparing homemade infant formula [13]; (3) introducing cow’s milk before one year of age [14]; and (4) using human milk from informal sharing [15]. In a recent prospective cross-sectional study of parents predominantly from low-income communities, the percent of individuals that used at least one unsafe infant feeding practice increased from 8% before to 48.5% during the 2022 infant formula shortage. Specifically, the percent of parents that reported infant feeding practices before and during the infant formula shortage significantly increased 5% to 26% for use of human milk from informal sharing and 2% to 29% for use of watered-down infant formula [16]. These unsafe infant feeding practices increase the health and safety risks of infants who depend on infant formula exclusively or as a supplement to human milk.

To date, no studies have reported the impact of the 2022 infant formula shortage on parental consumer behaviors, infant health or quality of life outcomes or breastfeeding outcomes. The purpose of this study was to identify infant feeding practices, health and quality of life outcomes during the 2022 infant formula shortage. We conducted a retrospective cross-sectional analysis and needs assessment in families from middle-high-income communities and reported a high percentage of infants who required specialty formulas. The goal of this study was to identify areas within regulatory and healthcare policies and programs that could improve the resiliency of the infant food system and prevent a future infant-feeding crisis. 

## 2. Materials and Methods

### 2.1. Subjects and Design

Parents who were signed up with the Bobbie Infant Formula (U.S.) listserv and agreed to be contacted for future research purposes were emailed an invitation to participate in an anonymous, cross-sectional, electronic survey. Individuals who met all study criteria completed the survey between 18 December 2022 and 31 January 2023. The first one hundred individuals who completed the survey received a USD 50 electronic gift card. Individuals were eligible to participate if they met the following criteria: (1) were 18 years old or older; (2) lived with their infants in the U.S. in May 2022; (3) were the parent of an infant aged 6 months or younger in May 2022; (4) their infant consumed some amount of infant formula before the May 2022 shortage; (5) experienced challenges with feeding their infant because of the infant formula shortage in May 2022; and (6) agreed that only one parent of one baby from the same household would complete the survey. The study was approved by the UC Davis Institutional Review Board (IRB ID: 1920147).

The online survey was created in Qualtrics 2022 (Provo, UT, USA) and consisted of ninety-four unique questions. Participants answered yes/no, multiple choice, rating on a sliding scale (0 to 10) and open-ended questions. The survey contained questions about demographics, infant feeding practices, experiences and sentiments in response to the infant formula shortage. Participants were asked what their infants typically ate over a 7-day period right before and a 7-day period during the infant formula shortage. Moreover, the use of human milk from informal sharing, homemade infant formula, watered-down formula and expired infant formula were aggregated into a single variable named “unsafe infant feeding practices” for a 7-day period right before and a 7-day period during the most challenging time of the shortage.

Participants were asked to select resources that provided guidance or support in feeding their infants during the shortage. Participants were also asked to rate on a sliding scale from 0 to 10 how helpful a list of resources “have been” with providing guidance or support to feed their infants or to select “non-applicable” if they did not receive any help from the listed resources. Infant feeding sources and resources used in this survey were adapted from the Infant Feeding Practices Study II (IFPS II) [17] which differentiated lactation consultants from other healthcare providers and included up-to-date online resources such as social media and blogs.

Participants were prompted to rate on a sliding scale ranging from 0 to 10 how helpful a list of activities “would be” in helping families feed their infants in the “near future” or to select “non-applicable” if uncertain. These activities were compiled from comments posted on several multiple social media outlets (Reddit, Facebook, Twitter) by parents during the peak of the 2022 infant formula shortage. The survey used in this study is available as Supplementary File S1.

### 2.2. Data Validation 

To ensure the reliability of the data collected from the survey and that it was not completed in duplicate by the same individuals, individuals completed two surveys. The first survey was used for screening and prompted individuals from the Bobbie Infant Formula listserv to answer questions about their eligibility. Individuals who met all eligibility criteria were emailed the cross-sectional survey and were required to use the same email addresses entered in the screening survey. The cross-sectional survey excluded individuals with the same email addresses. Only data or completed surveys were used in the statistical analyses. 

### 2.3. Statistics

All statistical analyses were conducted using IBM SPSS Statistics version 29 and figures were created in GraphPad PRISM v.10.1.2. Statistical significance was defined as *p* < 0.05. Bonferroni-adjusted *p*-values for multiple comparisons are reported herein. Descriptive statistics including means, standard deviations, ranges, frequencies, and percentages are reported for demographics, breastfeeding experience, infant feeding practices, consumer behavior, infant outcomes, use of and sentiments about resources and sentiments about future activities that could help families feed their infants in the future.

To understand how infant feeding changed in response to the infant formula crisis, parents were asked what their infants typically ate over a 7-day period right before and a 7-day period during the most challenging time of the infant formula shortage. Parents were also asked how they obtained formula before and during the shortage. Data were treated as binary (yes/no) responses and the McNemar test was used to determine if there were differences for each item before and during the infant formula shortage.

Parents were asked if their infants experienced any problems in response to changing infant formulas during the shortage. Parents selected problems from a list which were treated as binary (yes/no) responses and Pearson chi-square, 2-sided test and phi correlation were used to determine differences and their effect sizes, respectively, in the number of individuals whose infants experienced problems based on their requirement for specialty formulas (vs. no requirement for specialty formulas). 

The survey asked parents to choose all applicable options from a list of nineteen resources that provided guidance or support in feeding their infants during the infant formula shortage. Parents who selected “yes” to using any of the nineteen resources were asked to rate on a scale 0–10 how helpful those resources “have been” in providing guidance or support in feeding their infants during the infant formula shortage. Participants were also asked to rate on a scale 0–10 how helpful they think seventeen activities “would be” in helping families feed their babies in the “near future”. 

This study also collected qualitative data and prompted parents to offer open responses about how they dealt with the infant formula shortage and the actions they feel should be taken by health authorities, food companies and the government that could help them feed their infants during this crisis and prevent future crises. Open-ended responses were searched for repetition in 5% or more of respondents and reduced to thirty-eight words, themes or key phrases shown in Table S1. Canva^®^ 2023 version 1.82.0 was used to manually generate a word cloud of terms for which font size and color are proportional to the frequency of repeated words, themes or key phrases.

## 3. Results

### 3.1. Demographics and Participant Characteristics 

A total of 213 individuals initiated and 178 completed the survey. Respondents resided in thirty-seven unique U.S. states (Figure S1). All participants identified themselves as the parent (biological or not biological) of an infant who was six months old or younger in May 2022. Of parents who completed the survey, 93% were female and 77% were White, 12.4% were Asian, 0.6% were Black, 0.6% were American Indian or Alaska Native, 0.6% were unsure of their race and 3.4% reported belonging to two or more races. Approximately 88% of parents had earned a bachelor’s degree or higher and 55% of parents reported annual household incomes at or above USD 150,000/year. Six percent of parents reported that either they or their infants’ co-parent had received benefits from the Special Supplemental Nutrition Program for Women, Infants, and Children (WIC) within the past 12 months of completing the survey (Table 1).

Ninety-three percent of infants in this study joined their family through pregnancy. Eighty-nine percent of infants were born at term (≥37 weeks gestation) with an average age of 10.2 weeks at the time of the survey. Sixty-eight percent of female parents were primiparous when they completed the survey and ninety-six percent of female parents reported they had ever breastfed or fed expressed milk to their infants (Table 2). Of parents, 21% (*n* = 38) reported their infants required a specialty formula during the infant formula shortage due a metabolic or medical condition d for the following reasons: allergy (3.4%), colic (4.5%), constipation (5.6%), gas (8%), intolerance (1%), malabsorption (0.6%), prematurity (5.6%), rash (0.6%), reflux (6.2%) and other (3.4%) (Figure S2).

### 3.2. Infant-Feeding Practices before and during the Infant Formula Shortage

**Figure 1:**
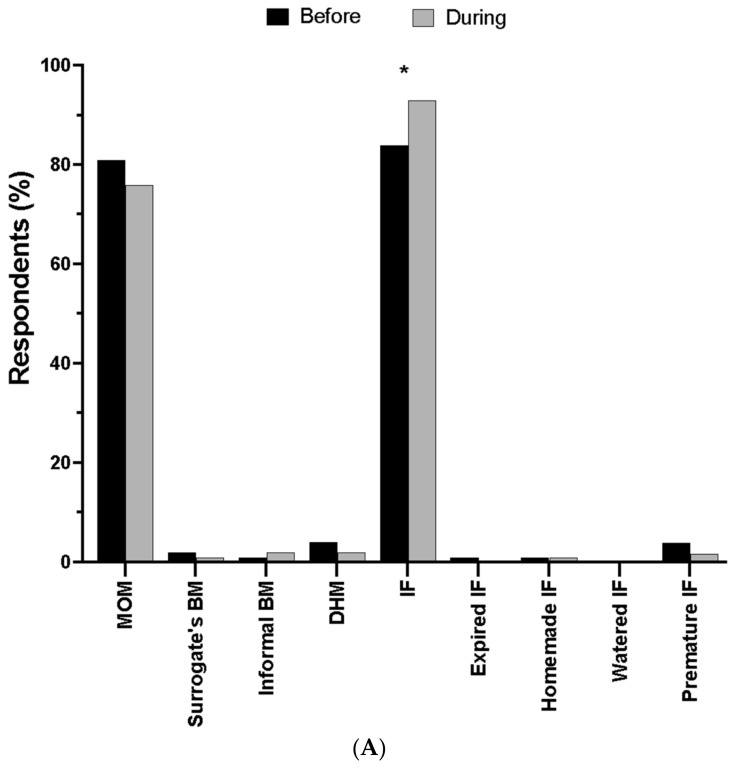

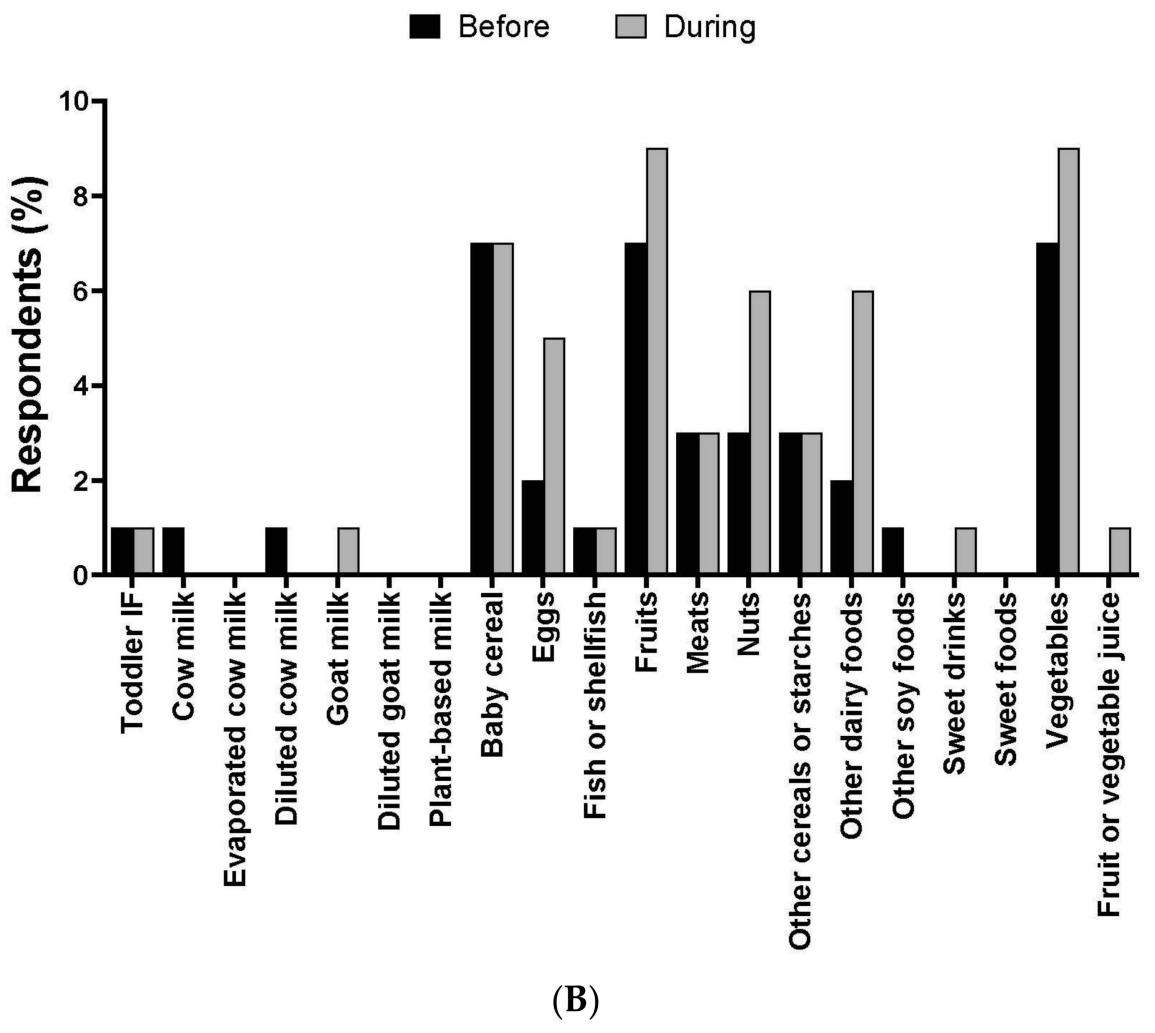
Infant-feeding practices before and during the infant formula shortage. (**A**) Human milk and infant formulas. MOM = mother’s own milk; BM = breast milk; DHM = donor human milk; IF = infant formula. * *p* < 0.01 (*n* =178). (**B**) Complementary foods and beverages. Data are expressed as the mean (*n* =178).

Prior to the shortage, 81% of infants in this study consumed their mothers’ own breast milk (MOM) but this value decreased to 76% during the infant formula shortage (trend, unadjusted *p* = 0.022). On the other hand, the use of infant formula significantly increased from 84% before the shortage to 93% during the shortage (adjusted *p* < 0.01) (Figure 1A). The use of any unsafe infant feeding practice, such as human milk from informal sharing, or homemade infant formula, or diluted formula, or expired infant formula, was low (2%) or did not change. The use of animal milks, plant-based milks, toddler formula, baby cereal or other foods or beverages was not significantly different, however, there was a trend for an increase in “other dairy foods, yogurt, cheese, ice cream, pudding, etc.” (trend, unadjusted *p* = 0.039) from 2% before to 6% during the infant formula shortage (Figure 1B). When asked how sure respondents were of their answers, 91% and 89% were “extremely sure” about questions about infant diet before and during the shortage, respectively.

**Figure 2:**
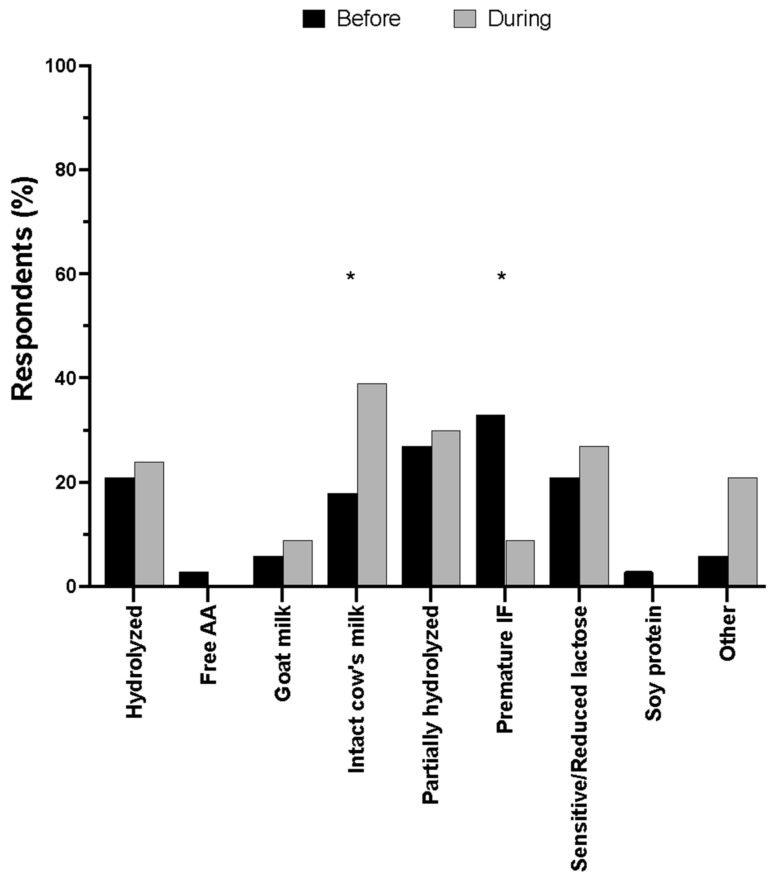
Type of specialty infant formula used before and during the infant formula shortage. AA = amino acids; IF = infant formula. * *p* < 0.05. Data are expressed as the mean (*n* = 33).

Eighty-nine percent of parents used U.S. infant formula brands before the shortage, however, this value decreased to seventy-nine percent during the shortage. Additionally, 2% of parents used imported infant formula before the shortage and this value increased to 7% during the shortage (Figure S3). Of the types of infant formulas used before and during the shortage, intact cow’s milk formula increased from 18% to 39% (trend, unadjusted *p* = 0.039) and premature formula significantly decreased from 33% to 9% (adjusted *p* < 0.05) (Figure 2).

### 3.3. Consumer Behavior and Experience in Response to the Infant Formula Shortage 

**Figure 3:**
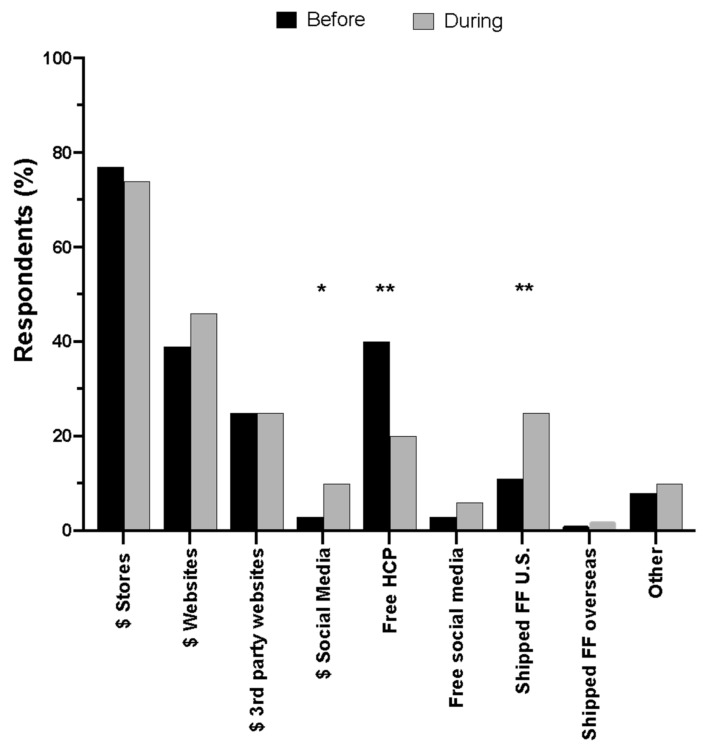
Methods used by parents to obtain infant formula before and during the infant formula shortage. HCP = healthcare provider; FF = friends or family. * *p* < 0.05; ** *p* < 0.01. Data are expressed as the mean (*n* = 178).

When asked how parents obtained infant formula, 3% purchased formula via social media before the shortage but this increased to 10% during the shortage (adjusted *p* < 0.05). The percent of parents that acquired free infant formula from healthcare providers significantly decreased from 40% before to 20% during the shortage (adjusted *p* < 0.01) but reliance on shipments of formula from friends or family within the U.S. significantly increased from 11% before to 25% (adjusted *p* < 0.01) (Figure 3).

Eighty-one percent of parents switched infant formulas during the shortage, and of these individuals, eighty-seven percent switched because they could not find the formula they “typically” used. Thirty-four percent of parents who switched infant formula brands or types during the shortage switched infant formulas three to five times. Twenty percent of parents reported having four weeks’ worth or more of infant formula at home during the infant formula shortage. Over a 24 h period, 29% of parents visited four or more stores and 26% of parents traveled more than twenty miles to one store to purchase infant formula. In this study, 13.9% of parents reported using Operation Fly during the shortage (Table S2). 

### 3.4. Infant Outcomes to the Infant Formula Shortage 

**Figure 4:**
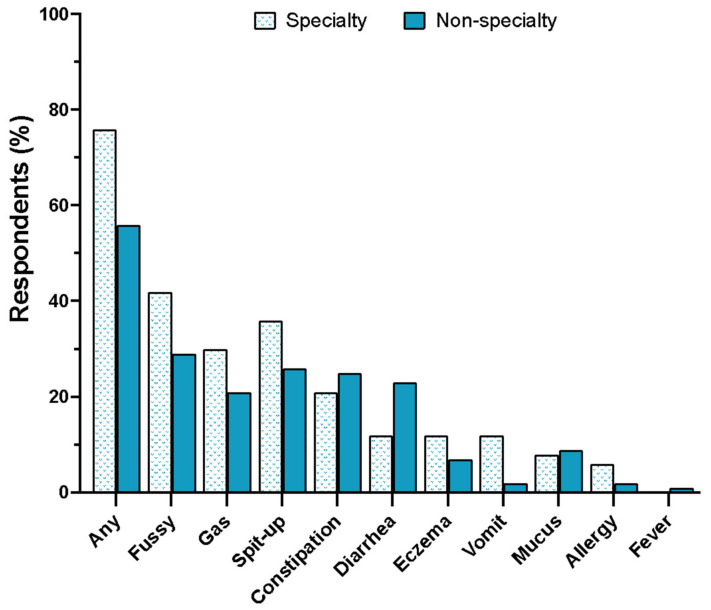
Health outcomes experienced by infants during the shortage. Infants that required specialty formulas (*n* = 32) vs. infants that did not require specialty formulas. Data are expressed as the mean (*n* =109).

Sixty-one percent of parents who switched formulas reported that their infants had one or more problems in response to switching. A sub-analysis revealed that infants requiring specialty formulas experienced more problems than infants who did not require specialty formulas. Compared with non-specialty formula users, more parents whose infants required specialty formulas reported their infants experienced “any” problems (unadjusted *p* = 0.045) and specifically vomiting (unadjusted *p* = 0.026) in response to switching formulas (Figure 4). 

### 3.5. Parental Use of Resources and Sentiments

**Figure 5:**
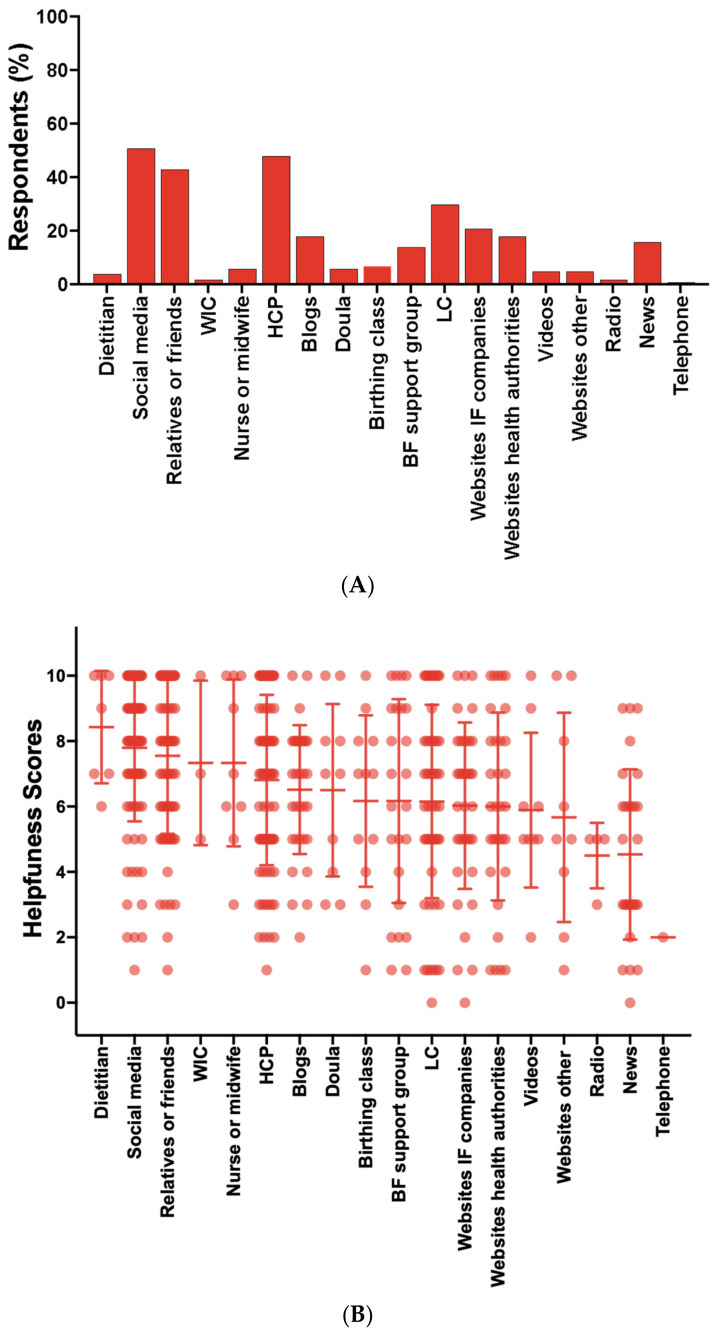
Resources parents used to obtain guidance or support to help them feed their infants during the infant formula shortage. (**A**). Percent of parents that used each resource. (**B**). Parents’ ratings on the helpfulness of each resource. Dietitian (*n* = 7); social media (*n* = 89); relatives or friends (*n* = 76); WIC (*n* = 3); nurse or midwife (*n* = 9); HCP (*n* = 84); blogs (*n* = 33); doula (*n* = 10); birthing class (*n* = 12); BF support group (*n* = 24); LC (n = 53); IF companies’ websites (*n* = 37); health authorities’ websites (*n* = 32); other websites (*n* = 9); videos (*n* = 9); other (*n* = 4); news (*n* = 28); radio (*n* = 4); telephone (*n* = 1). HCP = healthcare provider; BF = breastfeeding; LC = lactation consultant or lactation counselor; IF = infant formula. Data are expressed as the mean ± standard deviation error bars.

The most used resources used by parents for support and guidance in feeding their infants during the shortage were the following: social media (51%), healthcare providers (48%), relatives or friends (43%), lactation consultant or lactation counselor (30%), infant formula companies’ websites (21%), blogs (18%), health authorities’ websites (18%), media (news or magazines including TV broadcast, online articles) (16%) and breastfeeding support groups (14%) (Figure 5A). Parents’ mean ratings for the helpfulness of each resource were: social media (7.8), healthcare providers (6.8), relatives or friends (7.6), lactation consultant or lactation counselor (6.2), infant formula companies’ websites (6.0), blogs (6.5), health authorities’ websites (6.0), media (4.5) and breastfeeding support groups (6.2) (Figure 5B). Of the parents that used healthcare providers for support, 27% received recommendations to switch formulas, 25% received formula samples and 16% received information about lactation and breastfeeding from their infant’s healthcare provider (Figure S4). 

**Figure 6:**
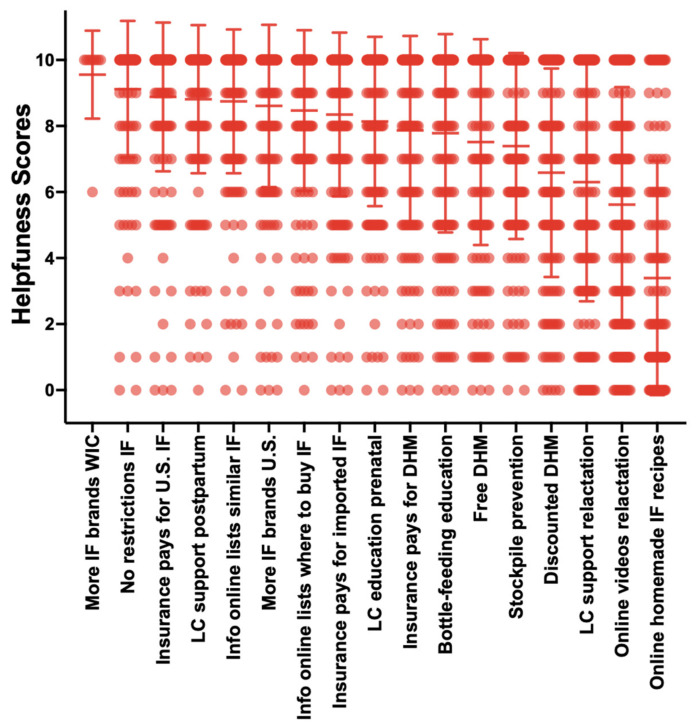
Parents’ ratings on the helpfulness of activities that would help families feed their infants in the near future. More IF brands in WIC (*n* = 9); no restrictions on IF (*n* = 166); insurance pays for U.S. IF (*n* = 169); LC support postpartum (*n* = 169); info online of lists of similar IF (*n* = 171); more IF brands sold in U.S. (*n* =166); info online of lists of where to buy IF (*n* = 171); insurance pays for imported IF (*n* =167); prenatal LC education (*n* = 168); insurance pays for DHM (*n* = 152); bottle-feeding education (*n* = 169); free DHM (*n* = 148); stockpile prevention (*n* = 163); discounted DHM (*n* = 140); LC support with relactation (*n* = 157); online videos of relactation (n = 158); online homemade IF recipes (*n* = 154). IF = infant formula; LC = lactation consultant or lactation counselor; DHM = donor human milk. Data are expressed as the mean ± standard deviation error bars.

To assess parents’ unmet needs, they were asked to rate the potential helpfulness of activities that would help families feed their infants in the “near future”. The highest mean scores for these future activities include: more infant formula brands that are available through WIC (9.6); parents can choose any infant formula brand without restrictions (9.1); health insurance and Medicare pay for all U.S. brands (8.9) and imported brands (8.4) of infant formula; information is available online that describes formula brands that meet infants’ unique health needs (8.8) and infant formula availability in stores (8.5); women receive free postpartum lactation support (8.8) and free prenatal lactation education (8.1) (Figure 6).

**Figure 7:**
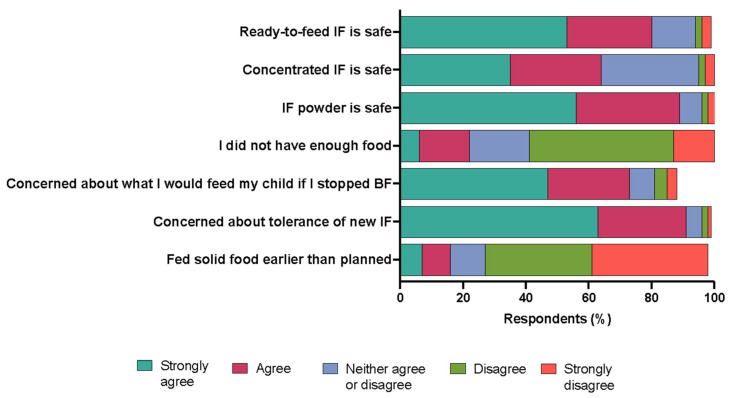
Parents’ perceptions of and relation to statements about infant formula. IF = infant formula; BF = breastfeeding. Data are expressed as the mean (*n* = 178).

Regarding parents’ perceptions about the safety and quality of infant formula, the following percentages of parents agreed or strongly agreed with these statements: ready-to-feed infant formula is safe for infants (80%), concentrated liquid infant formula is safe for infants (64%) and infant formula powder is safe for infants (89%). When asked how they related to some situations associated with the infant formula shortage, the following percentages of parents agreed or strongly agreed with these statements: they did not have enough food to feed their infants (22%), concerned about what they would feed their infants if they stopped breastfeeding (83%), concerned about how their infants would tolerate a new infant formula that was available (91%), introduced solid food to their infants earlier than initially planned (16%) (Figure 7).

### 3.6. Breastfeeding Goals, Experience and Support

Because exclusive human milk feeding is recommended for infants during the first six months of life, female parents were asked questions about their breastfeeding goals, experience and support. Nearly 81% of female parents had plans to exclusively breastfeed and 87% of female parents reported that they did not “exclusively” breastfeed as long as they had planned (Table S3). 

With respect to breastfeeding support, 86.3% of female parents received breastfeeding support from a lactation professional within 72 h postpartum and 18% of these parents paid out-of-pocket for lactation support services. With respect to receiving prenatal and early postpartum lactation support, 49% of female parents reported that they participated in a prenatal breastfeeding support group or class and 35% of female parents reported that they participated in a postpartum breastfeeding support group or class (Table S3). Parents’ mean ratings for helpfulness of prenatal and postpartum breastfeeding support groups or classes were 5.5 and 6.7, respectively (Figure S5). Regarding the unmet breastfeeding support needs of parents who did not participate in prenatal or postpartum breastfeeding support groups/classes, free prenatal and postpartum breastfeeding support groups or classes were scored 7.5 and 7.9, respectively, for their potential helpfulness (Figure S6). 

### 3.7. Non-Structured Qualitative Assessment

This study collected qualitative data in an open-ended question that asked respondents how they dealt with the infant formula shortage and the actions they feel should be taken by health authorities, food companies and the government that could help them feed their infants during this crisis and prevent future crises (Table S4). Open-ended responses were shared by 124 parents and converted into a word cloud. The following were the most frequently used word cloud terms in descending order: formula, breastfeeding, pumping, prevent, stressful, access, imported, impaired choice, breastfeeding challenges and low milk supply (Figure S7).

## 4. Discussion

The 2022 infant formula shortage was an unprecedented infant-feeding crisis that led to nationwide food and nutrition insecurity in our most vulnerable population [7]. This study used a semi-structured questionnaire to investigate infant-feeding practices, parents’ consumer behaviors and infant outcomes in response to the shortage to identify areas within government, regulatory and healthcare systems and policies that could result in a resilient infant food system. 

The use of unsafe infant-feeding practices was low in this population and did not change in response to the infant formula shortage. These findings contrast with Cernioglo et al. who reported a significant increase in the use of any unsafe infant-feeding practice from 8% before to 50% during the infant formula shortage [16]. The differences in the use of unsafe infant-feeding practices between Cernioglo et al. and our study may reflect the differences between the two populations’ socioeconomic statuses and are supported by Marino et al., who reported higher use of unsafe infant-feeding practices by families from lower-income compared with higher-income communities [18]. 

While the use of cow milk, goat milk and alternative milk beverages was low (<1%) and did not change, other complementary foods such as baby cereal were used by 7% of parents before and during the shortage, and the use of other dairy such as yogurt increased from 2% before to 6% during the shortage. These data align with a cross-sectional analysis by Marino and co-workers showing 10% of infant formula users added cereal to formula and 7% fed their infants with solid foods instead of feeding infant formula [18]. 

The use of pasteurized donor human milk was low (2–4%) and did not significantly change in response to the shortage. These results differ from a recent cross-sectional study by DiMaggio and colleagues, which surveyed 2315 individuals and found that 8% of participants reported using donor milk [19]. However, our data are consistent with a generally low use of pasteurized donor human milk, which is largely reserved for premature and very-low-birthweight infants, in mostly term and healthy infants. 

Approximately 80% of infants were combination feeders (mother’s own breast milk and formula) and, unexpectedly, use of infant formula significantly increased during the shortage when out-of-stock rates climbed as high as 90% in some states. The unexpected increased use of formula during the shortage in this sample may be explained in part by the use of Operation Fly, increased purchases of infant formula via social media and increased shipments of formula sent from family and friends in the United States. Our observations are consistent with a recent qualitative study that reported white female parents expressed positive feelings for having supportive family members and friends who assisted them to find formula which was not described by any Black or SNAP- or WIC-eligible participants [20]. Another explanation for the increased use of infant formula during the shortage may be the relatively high rate of stockpiling. About 20% of parents stockpiled infant formula during the shortage with 4 weeks’ worth or more of infant formula at home. The American Academy of Pediatrics recommends purchasing no more than a 10-day to 2-week supply of formula to prevent hoarding [21]. Moreover, women had experienced physical or mental challenges with breastfeeding with 80.5% intending to exclusively breastfeed and nearly 90% did not meet their goals.

Based on consumer behavior assessments, parents experienced burden in navigating the May 2022 infant formula shortage. Approximately 80% of parents switched infant formula types or brands during the shortage, and of these individuals, 87% switched because they could not find the formula they “typically” used. One-third or more of parents switched infant formula brands or types three to five times and over a 24 h period visited four or more stores and traveled more than twenty miles to one store to purchase infant formula. These data are similar to a recent report from an online survey of 1070 U.S. consumers, of which one-third had tried to purchase formula during the May 2022 infant formula shortage and, of those consumers, 30% reported purchasing formula at multiple stores [22]. Another study by Sylvetsky and colleagues reported that parents spent hours searching for formula by driving from store to store and searching online during the May 2022 infant formula shortage [20]. These data identify a high burden to parents and potentially those from low-income communities for which transportation and time are barriers in accessing food [23]. 

Switching infant formula brands and types reduced infant quality of life, especially in infants that relied on specialty formulas. Approximately 60% of parents who switched formulas reported that their infants had one or more problems in response to switching infant formulas. In a sub-group analysis, the number of infants that experienced problems in response to switching formulas was higher in infants requiring specialty formulas compared with infants that did not. These data may be explained in part by the increased use of intact cow’s milk formula during the shortage. These observations are supported by Marino et al., who found two-thirds of parents whose infants relied on specialty formulas reported difficulties accessing these formulas during the COVID-19 pandemic [18].

Parents relied on several resources to navigate the infant formula shortage with social media and healthcare providers (50%) as the most used, followed by relatives or friends (43%), and about one-third of parents used a lactation consultant or lactation counselor which was lower than data from the CDC and FDA’s 2006 Infant Feeding Practices Study II (IFPSII) [17]. The mean helpfulness scores for these resources were moderate with social media scoring the highest. This is consistent with findings from other studies that highlight social media as a powerful tool for diet and health education and support [16,20]. Parents’ mean helpfulness scores for future activities that would facilitate feeding infants in the “near future” included freedom to choose infant formula brands, health insurance coverage for infant formula, online resources describing formula types or brands that meet infants’ unique health needs and free universal prenatal lactation education and postpartum lactation support. 

Because exclusive human milk feeding is recommended for infants during the first six months of life, female parents were asked questions about their breastfeeding goals, experience and support. Most (80.5%) female parents planned to exclusively breastfeed their infants, however, 87% did not meet their exclusive breastfeeding goals. These data are fairly consistent with the CDC Breastfeeding Report Card showing most U.S. infants (84.1%) have ever consumed any breast milk but only 25% of infants meet the national recommendations of exclusive breastfeeding through six months of life [6]. There are several factors that explain why women are unable to meet their breastfeeding goals, from lack of federal paid family and medical leave policies, insufficient flexibility and privacy for mothers to breastfeed or pump while at work to barriers in affording or accessing prenatal lactation education and postpartum lactation support which are not part of standard care [24,25,26]. 

This is the first report of infant-feeding outcomes and consumer behavior in response to the May 2022 infant formula shortage with different limitations and strengths. The limitations include the study design which was 7–8 months retrospectively and is at risk for high recall errors. This may explain in part the small number of parents reporting use of unsafe infant-feeding practices compared to a more recent cross-sectional study in a similar population [19]. Second, our target population included parents who subscribed to the Bobbie Infant Formula listserv which may restrict the generalizability of the findings. Nearly 80% of respondents were derived from largely White communities with annual household incomes equal to or greater than USD 100,000 and not representative of U.S. families that were severely affected by the infant formula shortage. Notably, our survey failed to capture sufficient responses from individuals who represent families from low-income, Black and Hispanic communities that were hit the hardest by the shortage [10]. There are also several strengths of this study. First, data from this cross-sectional study were collected from a large sample of parents who resided across the United States which improves the study’s generalizability. Second, nearly 90% of respondents reported being “extremely sure” about their answers regarding their infant’s feeding practices before and during the shortage, demonstrating potentially low recall biases. Additionally, 70% of parents answered the open-response question which suggests high engagement with the survey and a proxy for high-quality data. Finally, the survey collected a combination of quantitative and qualitative data on infant feeding, parental experiences and sentiments and infant outcomes that could identify areas in policy and educational strategies to assist families in averting future infant-feeding crises. 

We propose a call to action for government, regulatory, health and workplace policies that prioritize infant-feeding practices that deliver optimal nutrition, safety and food security. First, the U.S. infant formula supply is controlled by U.S. trade and regulatory policies that result in a U.S. infant formula monopoly [27]. High tariffs on formula thwart the import of infant formula to the U.S. and federal policies that govern the manufacture and labeling of infant formula exclude it from entering the U.S. legally [4]. Systemic failures that reduce infant formula diversification and support a monopoly inequitably impacted low-income communities such as WIC recipients and nutritionally vulnerable infants [4]. There is a temporary solution with recent modifications to the Access to Baby Formula Act that became law in February 2024. This amendment to the Child Nutrition Act of 1966 will develop a waiver authority to address emergencies, disasters and supply chain disruptions by ensuring WIC state offices can secure supplies from additional manufacturers outside of their contracts. Yet, this law is a stopgap and prevention of another feeding crisis will depend on systemic changes to healthcare policies that also protect the infant-feeding system with access to banked donor milk and lactation education and support. The accessibility and growth of donor-milk-banking services are hindered in part by a lack of federal public health policies that integrate donor milk banking or regulate its operations. Finally, the U.S. government and healthcare system should commit to implementing policies that prioritize lactation education and support. The suboptimal breastfeeding rates in the U.S. and within low-income communities are a result of a lack of federal paid family and medical leave; the absence of flexibility and privacy for mothers to breastfeed or pump while at work; and difficulty affording lactation services, which are not part of standard care. The future of individual, community and societal health relies on optimal early life nutrition that is resilient and equitable for all.

## 5. Conclusions

This study is the first to report infant outcomes, parental consumer behavior and needs in infant feeding in response to the 2022 infant formula shortage. Most parents in this study switched infant formula brands or types because they could not find the formula they typically used. One-third of parents who switched formulas had changed formula brand or type multiple times and frequently visited stores and traveled long distances to purchase formulas. Infant formula use increased in response to the infant formula shortage which suggests this population had resources and support unlike families from low-income communities that used unsafe infant-feeding practices. The most vulnerable infants who required specialty formulas had experienced more problems switching formulas. While parents used social media, healthcare providers and relatives/friends for support during the shortage, these resources’ helpfulness scores were suboptimal. Parents reported that wide availability of infant formula options, infant formula covered by insurance without restrictions, free, universal prenatal lactation education and postpartum lactation support as solutions to the crisis. Our report highlights barriers in safely feeding infants and presents potential health disparities in families despite their economic status. Our results demonstrate that systemic changes to policies that diversify and fortify the infant food supply, ensure equitable and reliable access to banked donor milk and integrate universal prenatal and postnatal breastfeeding support in healthcare are critical to protect the most vulnerable population from another feeding crisis.

## Figures and Tables

**Table 1:** Parental characteristics.

Parental Sex, % (*n*)	
Female	93.3% (166)
Male	5.1% (9)
Decline	1.7% (3)
Ethnicity, % (*n*)	
Hispanic	10.7% (19)
Non-Hispanic	87.1% (155)
Decline	2.2% (4)
Race, % (*n*)	
White	77% (137)
Asian	12.4% (22)
Black/African American	0.6% (1)
American Indian/Alaska Native	0.6% (1)
Unsure	0.6% (1)
2 or more	3.4% (6)
Decline	3.9% (7)
Other	1.7% (3)
Education, % (*n*)	
High School	1.7% (3)
Some college	2.2% (4)
Associate’s degree	8.4% (15)
Bachelor’s degree	44.4% (79)
Master’s degree	30.9% (55)
Professional or doctorate	12.4% (22)
Marital status, % (*n*)	
Married/unmarried couple	92.7% (165)
Divorced/separated	1.1% (2)
Never married	6.2% (11)
Household size (people), % (*n*)	
1	0.6% (1)
2	0.6% (1)
3	57.9% (103)
4	29.2% (52)
5	10.1% (18)
6	0.6% (1)
7	1.1% (2)
Household income, % (*n*)	
Less than USD 25,000	1.1% (2)
USD 25,000–USD 34,999	1.1% (2)
USD 35,000–USD 49,999	1.7% (3)
USD 50,000–USD 74,999	5.6% (10)
USD 75,000–USD 99,999	7.3% (13)
USD 100,000–USD 149,999	23% (41)
USD 150,000–USD 199,999	20.2% (36)
More than USD 200,000	34.3% (61)
Unsure	1.1% (2)
Decline	4.5% (8)
Receiving WIC benefits, % (*n*)	
Yes	6.2% (11)
No	93.3% (166)
Decline	0.6% (1)

**Table 2:** Infant characteristics and female parental reproductive history.

	Mean ± SD	Range (Weeks)
Infant age	10.2 ± 6.8	0.29–24.7
Number of pregnancies, % (*n*) ^1^		
0	1.2% (2)	
1	52.4% (87)	
2	24.7% (41)	
3	12.0% (20)	
4	4.2% (7)	
5	3.0% (5)	
6	1.2% (2)	
7	1.2% (2)	
Parity, % (*n*) ^1^		
Primiparous	68% (111)	
Multiparous	32% (53)	
Infant relationship, % (*n*)		
Pregnancy	92.7% (165)	
Partner pregnancy	5.1% (9)	
Surrogacy	0.6% (1)	
Adoption	1.1% (2)	
Decline	0.6% (1)	
Multiple births, % (*n*)		
Single	96.1% (171)	
Twins	3.9% (7)	
Infant gestational age at birth, % (*n*)		
Term	89.3% (159)	
Preterm	10.7% (19)	
Ever breastfed, % (*n*) ^2^		
Yes	96.4% (159)	
No	3.6% (6)	
Delivery location, % (*n*) ^3^		
Birth center	3.1% (5)	
Hospital	96.3% (154)	
Other	0.6% (1)	
Delivery mode, % (*n*) ^3^		
C-section emergent	14.4% (23)	
C-section scheduled	21.3% (32)	
Vaginal	64.4% (103)	
Infants transferred to NICU, % (*n*) ^3^		
Yes	13.1% (21)	
No	86.9% (139)	
Skin to skin contact, % (*n*) ^3^		
Yes	89.4% (143)	
No	8.8% (14)	
Unsure	1.3% (2)	
Decline	0.6% (1)	
Infant fed 72 h postnatal, % (*n*)		
Mom’s breast milk ^3^		
Yes	89.4% (143)	
No	10.6% (17)	
Donor human milk ^3^		
Yes	9.4% (15)	
No	90.6% (145)	
Infant formula ^3^		
Yes	52.5% (84)	
No	47.5% (76)	
Other ^3^		
Yes	1.9% (3)	
No	98.1% (157)	

^1^ Twelve respondents did not complete this question. ^2^ Thirteen respondents did not complete this question. ^3^ Eighteen respondents did not complete this question.

## Data Availability

The original contributions presented in the study are included in the article/Supplementary Materials, further inquiries can be directed to the corresponding author/s.

## Supplementary Materials

The following supporting information can be downloaded at: https://www.mdpi.com/article/10.3390/nu16050748/s1, Figure S1: Map of parental residence across thirty-seven states; Figure S2: Percentage of parents that reported reasons their infants required specialty formulas; Figure S3: Manufacturing locations of typical formulas consumed by infants before and during the infant formula shortage; Figure S4: Guidance or support provided by healthcare providers to parents during the infant formula shortage; Figure S5: Scores in helping female parents achieve their breastfeeding goals with prenatal and postpartum breastfeeding classes or support groups that they had completed; Figure S6: Scores in helping women achieve their breastfeeding goals with future free prenatal and postpartum breastfeeding classes or support groups; Figure S7: Word cloud that summarizes parents’ open-ended responses; Table S1: Words, themes and key phrases used to generate a word cloud; Table S2: Practices carried out by parents in response to the infant formula shortage; Table S3: Breastfeeding experience and goals; Table S4: Open-ended responses by participants.
