# Supplementary material for: Cross-Sectional Analysis of Infant Diet, Outcomes, Consumer Behavior and Parental Perspectives to Optimize Infant Feeding in Response to the 2022 U.S. Infant Formula Shortage
Source: Nutrients. 2024 Mar 5;16(5):748. doi: 10.3390/nu16050748 (PMC10934383; doi:10.3390/nu16050748)
Supplement: Supplementary file 1 [file nutrients-16-00748-s001.zip › nutrients-2866736-supplementary.pdf]

## Supplementary Material

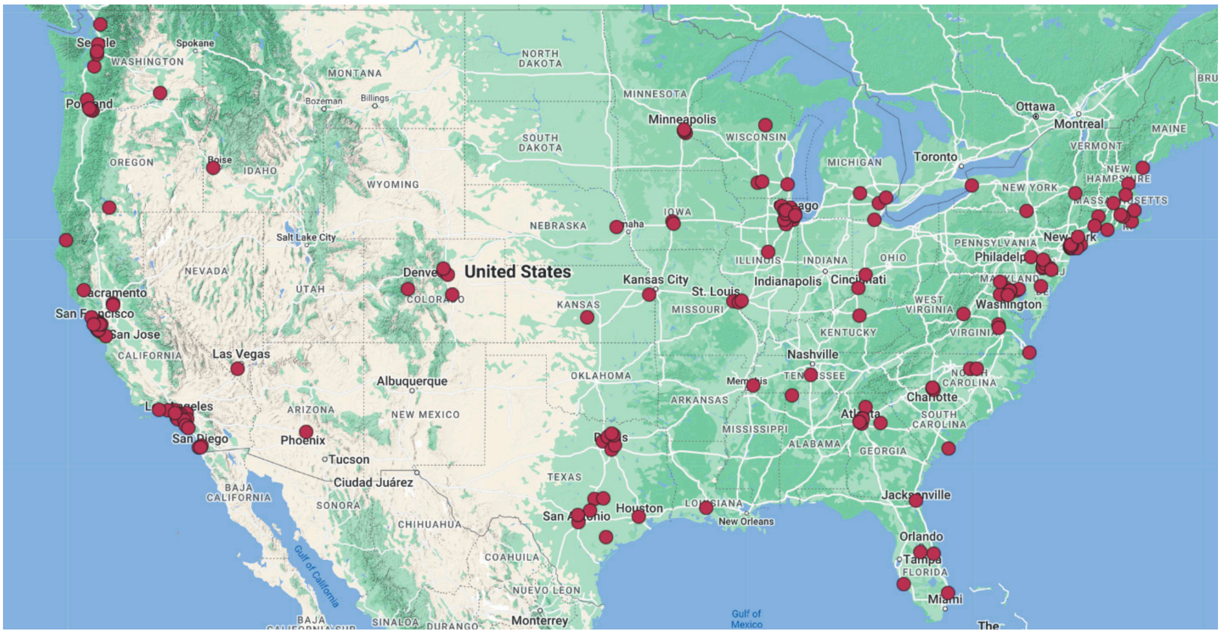


**Figure S1.** Map of parental residence across thirty-seven states.

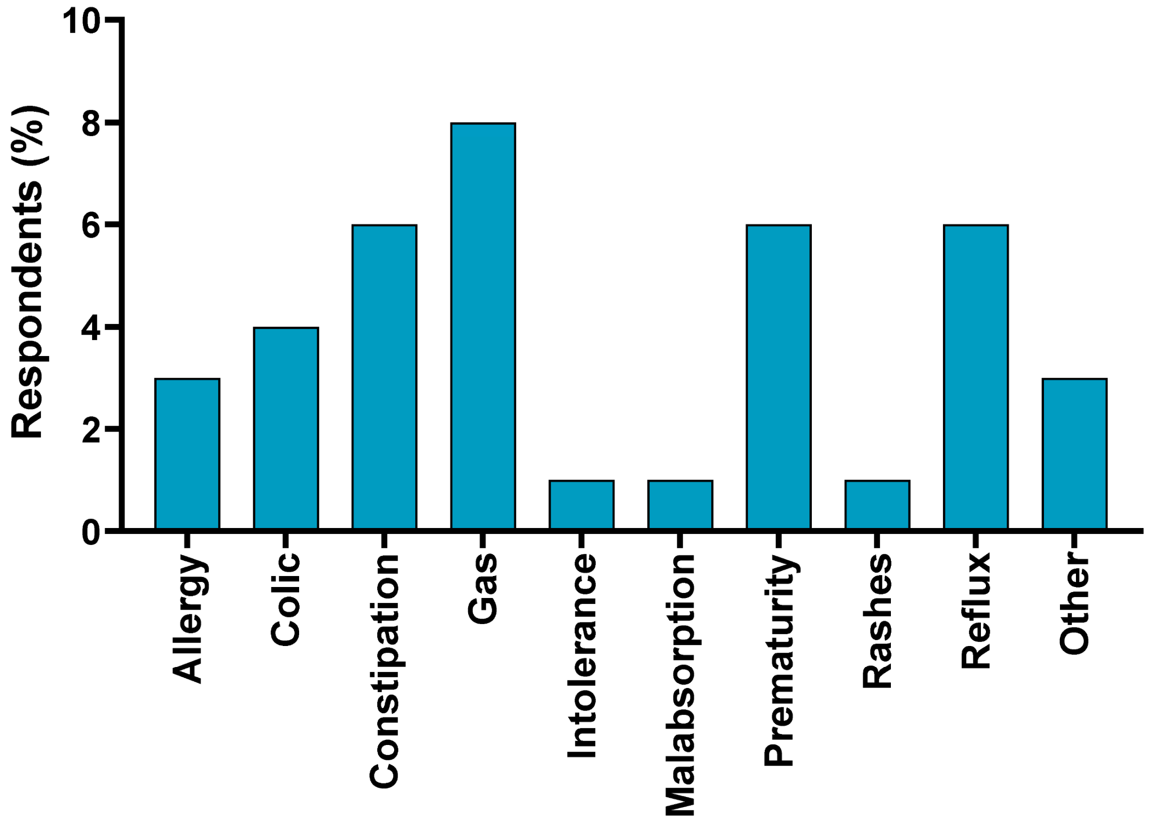


**Figure S2.** Percentage of parents that reported reasons their infants required specialty formulas. Allergy (n = 6); colic (n = 8); constipation (n =10); gas (n = 14); intolerance (n = 2); malabsorption (n = 1); prematurity (n = 10); rashes (n = 1); reflux (n = 11); other (n = 6). Data are expressed as the mean.

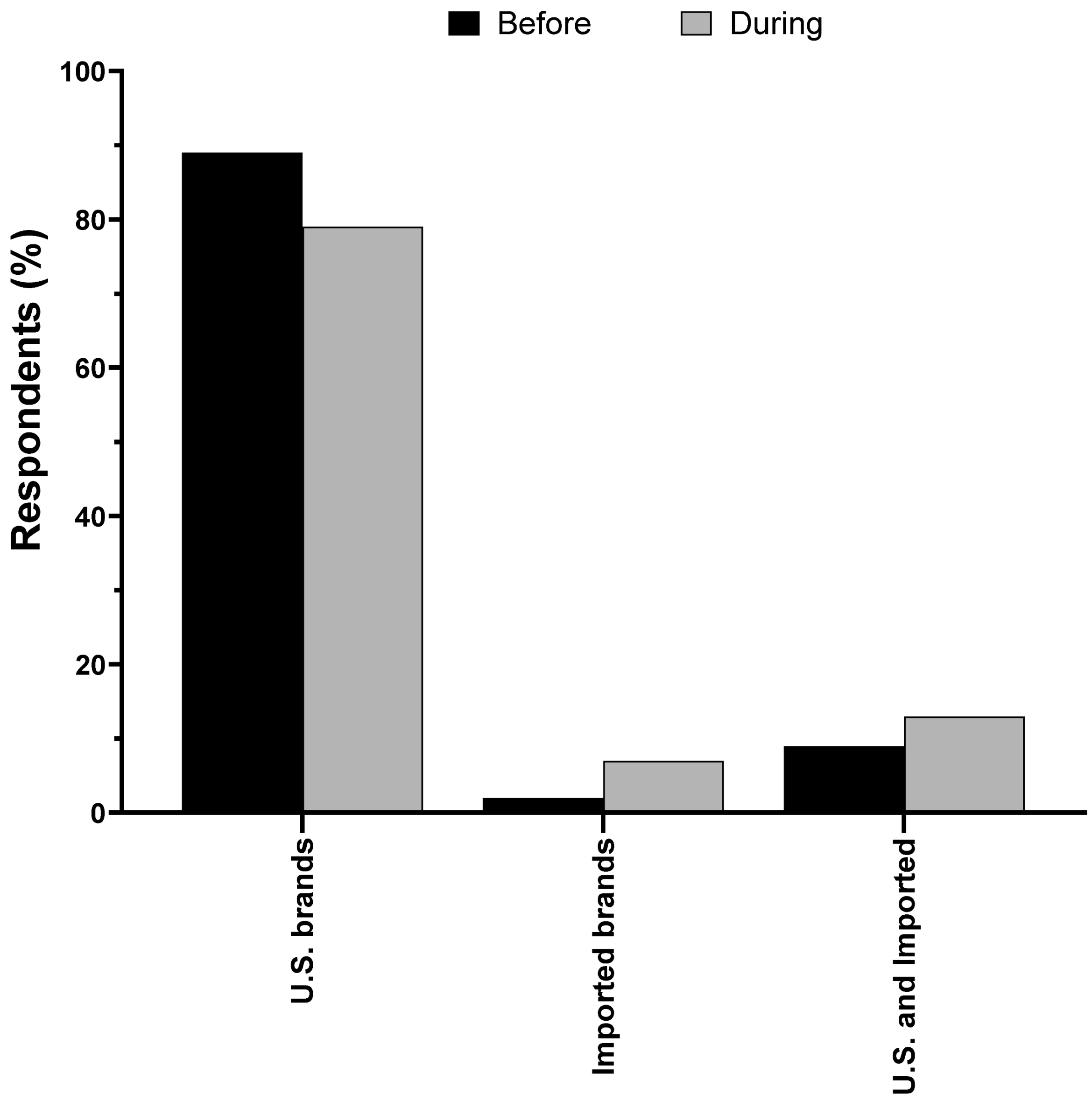


**Figure S3.** Manufacturing locations of typical formulas consumed by infants before (n = 149) and during (n = 164) the infant formula shortage. Data are expressed as the mean.

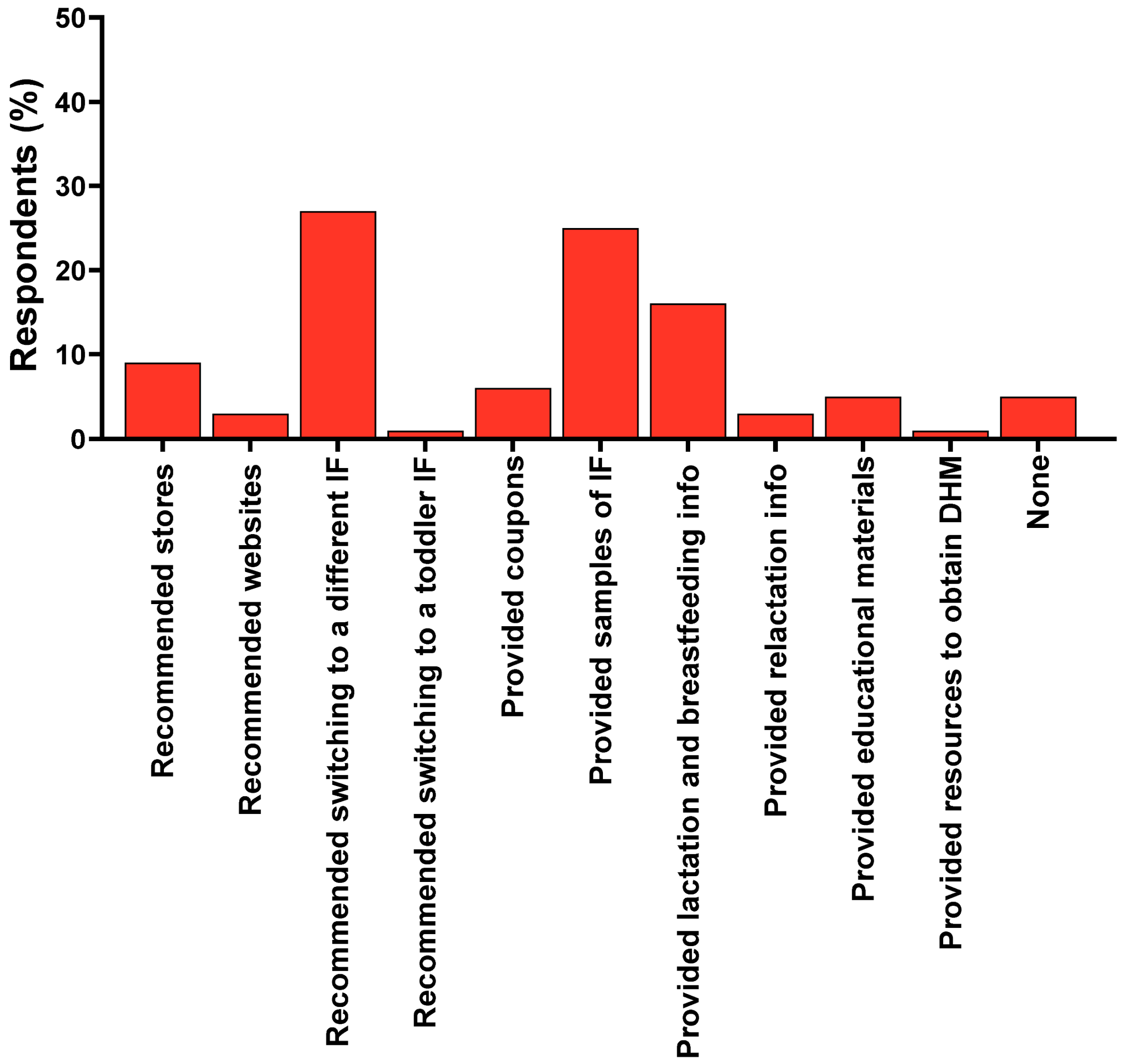


**Figure S4**. Guidance or support provided by healthcare providers to parents during the infant formula shortage. Recommended stores (n = 16); recommended websites (n = 5); recommended switching to a different IF (n = 48); recommended switching to a toddler formula (n = 1); provided coupons (n = 10); provided samples of IF (n = 45); provided lactation and breastfeeding info (n = 29); provided relactation info (n = 6); provided educational materials (n = 8); provided resources to obtain DHM (n = 1). IF = infant formula; DHM = donor human milk; info = information. Data are expressed as the mean.

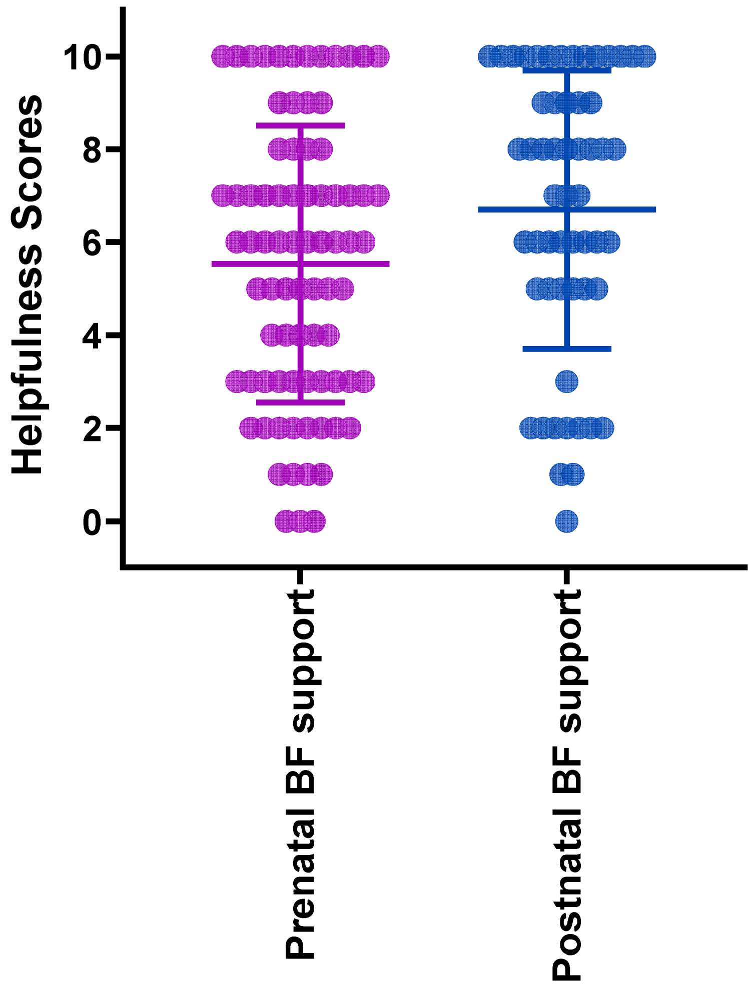


**Figure S5**. Scores in helping female parents achieve their breastfeeding goals with prenatal (n = 79) and postpartum (n = 56) breastfeeding classes or support groups that they had completed. Data are expressed as the mean ± standard deviation error bars.

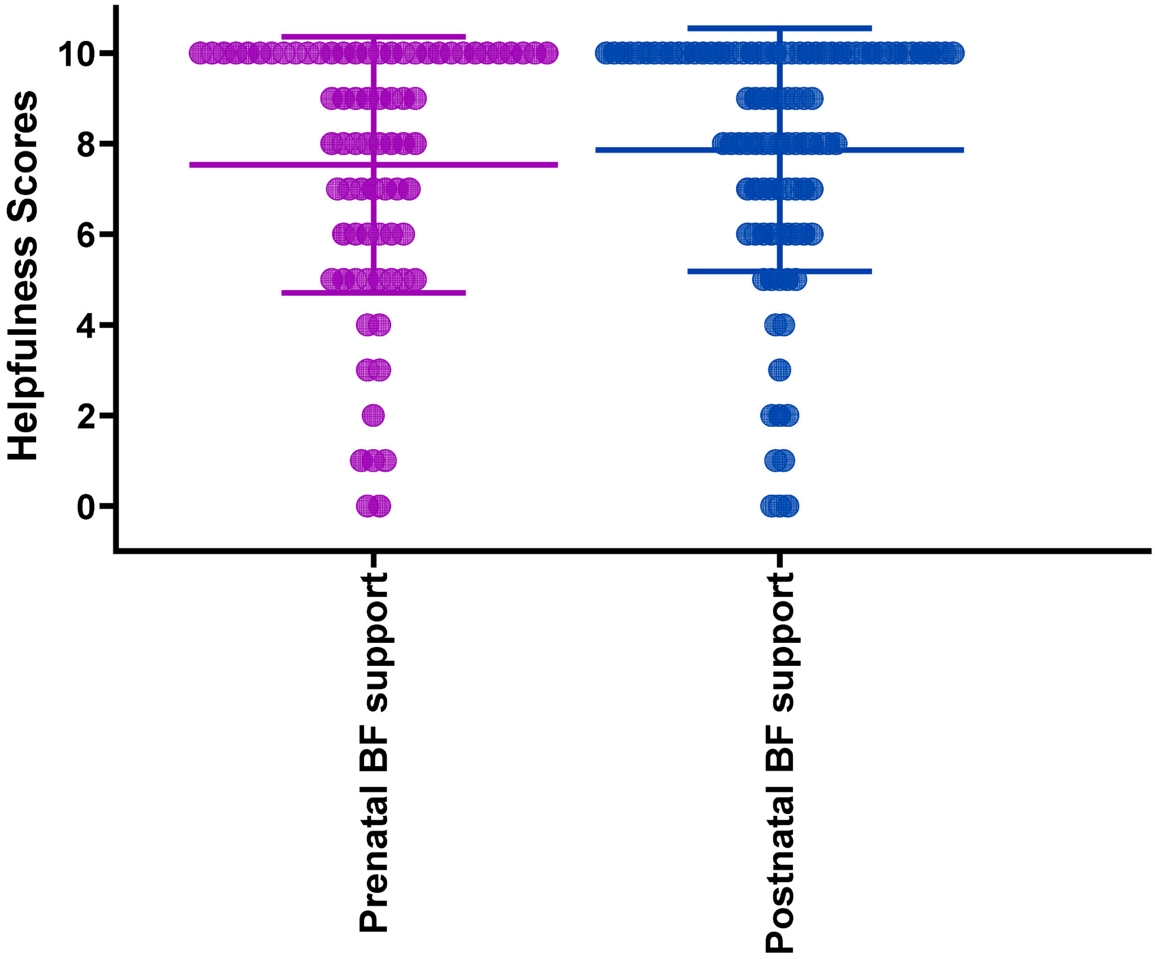


**Figure S6.** Scores in helping women achieve their breastfeeding goals with future free prenatal (n = 77) and postpartum (n = 102) breastfeeding classes or support groups. Data are expressed as the mean ± standard deviation error bars.

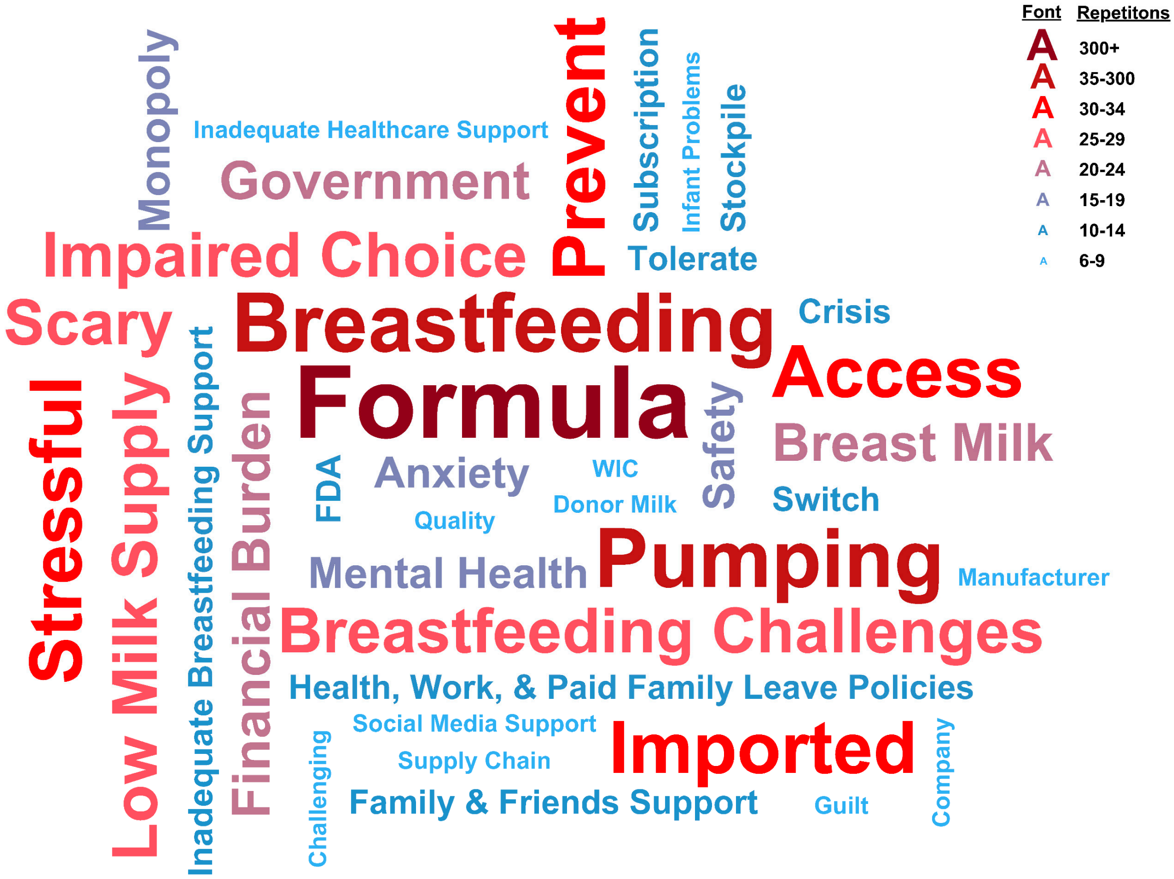


**Figure S7.** Word cloud that summarizes parents' open-ended responses.

**Table S1**: Words, themes and key phrases used to generate a word cloud.

| Word cloud terms | # Repetitions | Words, themes or key phrases grouped with each word cloud term |
|---|---|---|
| Formula | 308 | Formula |
| Breastfeeding | 89 | Breastfeeding, breastfeed, breastfed |
| Pumping | 60 | Pumping, pumped, pump |
| Prevent | 34 | Preventable, could have been avoided, proposed methods to prevent in future |
| Stressful | 34 | Stressful, stress, trauma, traumatic |
| Access | 33 | Explained difficulty finding formulas or described amount of time it took to find formula or use of the words: travel, drove, drive, shipping |
| Imported | 31 | International, foreign countries, import, importation, imported |
| Impaired Choice | 29 | Parent started a feeding regimen outside of their plans, breastfed when they did not desire to, could not find the type of formula they needed, started solids sooner than planned, better tolerated formulas from imported companies, desire for more options |
| Breastfeeding Challenges | 26 | Difficulties breastfeeding unrelated to low breast milk supply or inadequate support, physically unable to breastfeed or use of the words: triple feeding, mastitis |
| Low Milk Supply | 26 | Described low breast milk supply |
| Scary | 25 | Fear, feared, fearing, afraid, terrified |
| Breast Milk | 23 | Breast milk |

| | | |
|---|---|---|
| Government | 22 | Government |
| Financial Burden | 21 | Free, expensive, subsidies, subsidize, financial assistance, insurance, costly |
| Mental Health | 19 | Postpartum depression, postpartum anxiety, mentally hard, emotional, emotionally draining, mentally draining |
| Safety | 17 | Safe, safely, safety, safeguards, jeopardy, unsafe, improved regulations, safety standards |
| Monopoly | 16 | Monopolies, monopolized |
| Anxiety | 15 | Anxious, nervous, worry, worried |
| Family & Friends Support | 14 | Friends or family helped acquire feeding solutions |
| Subscription | 14 | Subscription |
| Switch | 14 | Changed formulas, switched, change |
| Crisis | 13 | Crisis |
| Inadequate Breastfeeding Support | 12 | Described inadequate support from a lactation consultant, breastfeeding support is/was needed and not provided, need or desire for breastfeeding education |
| Stockpile | 12 | Stockpiling, panic buying, hoarding, hoard |
| FDA | 11 | FDA, regulatory |
| Tolerate | 11 | Descriptions of how baby tolerated switching formula |

| | | |
|---|---|---|
| Health, Work, & Paid Family Leave Policies | 10 | Description of health policies, paid family leave or work policies or use of the words: policy, policy maker, health authorities |
| Inadequate Healthcare Support | 9 | Described receiving inadequate help from healthcare providers other than lactation consultants, lack of or inadequate support to infant formula feed, need or desire for formula feeding education |
| Manufacturer | 9 | Factory, manufacturer, facilities, producers |
| Social Media Support | 9 | Description of any social media support including blogs, Facebook, online support groups |
| Challenging | 8 | Challenge, challenging, struggled, struggle, hard, harder, difficult |
| Quality | 8 | Quality control, quality assurance, standards |
| Donor Milk | 7 | Described use of human milk from an individual other than the parent including milk obtained from a donor milk bank or informal milk sharing |
| Guilt | 7 | Guilt |
| Infant Problems | 7 | Described health conditions infants had before or in response to the shortage |
| Supply chain | 7 | Supply, supply chain |
| WIC | 7 | WIC, low income |
| Company | 6 | Company |

**Table S2.** Practices carried out by parents in response to the infant formula shortage.

| | | % | (n) |
|---|---|---|---|
| Switched IF brands or types | | | |
| | Yes | 80.8 | 143 |
| | No | 19.2 | 34 |
| Switched IF due to unavailability | | | |
| | Yes | 87.4 | 125 |
| | No | 11.2 | 16 |
| | Unsure | 1.4 | 2 |
| Times switched IF | | | |
| | 1-2 | 60.1 | 86 |
| | 3-5 | 34.3 | 49 |
| | >5 | 4.2 | 6 |
| | Unsure | 1.4 | 2 |
| Most amount of IF at home | | | |
| | 1-4 days | 5.8 | 10 |
| | 5-9 days | 26.0 | 45 |
| | 10 days-2 weeks | 27.2 | 47 |
| | 1-4 weeks | 20.2 | 35 |
| | 4-6 weeks | 11.0 | 19 |
| | >6 weeks | 9.8 | 17 |
| Most times visited stores to purchase IF | | | |
| | 1 | 20.2 | 35 |
| | 2 – 3 | 45.7 | 79 |
| | 4 – 6 | 20.2 | 35 |
| | 7 – 10 | 2.3 | 4 |
| | More than 10 | 6.4 | 11 |
| | Unsure | 5.2 | 9 |
| Farthest distance traveled to purchase IF | | | |
| | 0-5 miles | 17.9 | 31 |
| | 6-10 miles | 20.2 | 35 |
| | 11-15 miles | 17.3 | 30 |
| | 15-20 miles | 17.9 | 31 |
| | 21-25 miles | 5.8 | 10 |
| | 25-30 miles | 6.4 | 11 |
| | More than 30 miles | 13.9 | 24 |
| | Unsure | 0.6 | 1 |

| | | |
|---|---|---|
| Used IF from "Operation Fly" | | |
| Yes | 13.9 | 24 |
| No | 79.8 | 138 |
| Unsure | 6.4 | 11 |

IF = infant formula.

**Table S3.** Breastfeeding experience and goals.

| | | % | (n) |
|---|---|---|---|
| Plans to exclusively breastfeed | | | |
| | Yes | 80.5 | 124 |
| | No | 16.0 | 25 |
| | Unsure | 3.2 | 5 |
| Exclusively breastfed as long as planned | | | |
| | Yes | 12.9 | 16 |
| | No | 87.1 | 108 |
| | Unsure | 0.6 | 1 |
| Breastfed as long as planned | | | |
| | Yes | 15.8 | 18 |
| | No | 78.9 | 90 |
| | Unsure | 5.3 | 6 |
| Received BF support within 72h postpartum | | | |
| | Yes | 86.3 | 138 |
| | No | 11.3 | 18 |
| | Unsure | 1.3 | 2 |
| | Decline | 1.3 | 2 |
| Paid for breastfeeding support | | | |
| | Yes | 18.1 | 29 |
| | No | 65.0 | 104 |
| | Unsure | 3.1 | 5 |
| Reasons for not receiving BF support | | | |
| | LP was not available when needed | 5.6 | 10 |
| | I did not think I needed help | 0.6 | 1 |
| | I was not offered to meet with an LP | 5.1 | 9 |
| | It was too expensive | 0.6 | 1 |
| | It was not covered by my insurance | 0.6 | 1 |
| | Other | 2.8 | 5 |
| Participation in prenatal BF class or support group | | | |
| | Yes | 49.4 | 79 |
| | No | 50.0 | 80 |
| | Unsure | 0.6 | 1 |

| | | |
|---|---|---|
| Participation in postpartum BF class or support group | | |
| Yes | 35.0 | 56 |
| No | 64.4 | 103 |
| Unsure | 0.6 | 1 |
| Currently breastfeeding | | |
| Yes | 26.0 | 40 |
| No | 74.0 | 114 |

BF = breastfeeding; LP = lactation professional.

**Table S4.** Open-ended responses by parents.

| ID | **Please share your thoughts about how you have dealt with the infant formula shortage crisis and the actions you feel should be taken by health authorities, food companies, and the government that could help you feed your baby during this crisis and prevent future crises. Write "none" if you do not wish to leave a comment.** |
|---|---|
| 130 | I set out to exclusively breastfeed for a year but faced a number of challenges [with breastfeeding] early in my journey, including thrush and mastitis. I desperately wanted to stop breastfeeding but felt I couldn't because of the infant formula shortage. I developed a great deal of anxiety about feeding my baby. I was grateful to be able to breastfeed but also felt resentful that it didn't feel like a choice. I also became concerned about whether I would be able to continue breastfeeding when I returned to work (i.e., whether pumping would be successful). I had heard from other mothers whose breast milk supplies had dropped after retuning to work and felt I needed to have at least some formula on hand in case it became necessary. I simultaneously felt bad [guilt] about "taking" formula from parents who needed formula more immediately to feed their children. I felt like our country did not pay enough attention [ignored] to the issue until it was a crisis, and it has been far too quick to forget about the crisis without taking steps to prevent a recurrence. Based in everything I've read, health authorities and/or the government should have intervened much earlier [slow response] at the manufacturer that made the formula that was recalled. I also think policy makers need to figure out how to address what is essentially an infant formula monopoly. Infant formula should be regulated to ensure it's safe, but we need to be incentivizing and enabling competitors to enter the market. It's also ridiculous that programs such as WIC specify the type of infant formula families may buy for their children; among other things, it feels like an endorsement of a formula company that has a monopoly and was violating FDA standards. |
| 147 | It was ridiculous and scary not having options that I felt were healthy and safe for my child |
| 135 | I was thankful to have family searching in stores for me, and we never ran out (though we were afraid we would). I was also working with a lactation consultant throughout, but my breastmilk supply was never sufficient to feed my baby without supplementation. I was disappointed by the restrictions placed on european formulas, and felt a more diversified supply chain could have helped prevent this crisis. |
| 104 | My baby had a severe FPIES reaction to a partially hydrolyzed formula I had to use during the shortage and ended up in the ER. I am extremely angry that the government allowed the companies to slip in safety protocols (the FDA should be performing much more thorough inspections) and that the government allowed the situation where two companies control all of the formula in the US. It is unacceptable and just telling women to breastfeed ignores biological, social and environmental factors that make that an impossible task for many women. |
| 72 | None |
| 117 | I was following the news closely on recalls and had stockpiled some formula well in advance of the shortage. I ended up having to throw away some of that stockpile because it was recalled. I tried 4 different formulas because of the shortage, some of which caused issues with gas and it was stressful not knowing whether I'd be able to get the formula my baby tolerated best. Also, I was having issues with breastfeeding, which is already an emotional thing, and then feeling pressure to continue doing it because of the shortage was just an awful feeling. All around it just made an already stressful and chaotic time even more so. |
| 96 | I would have stopped breastfeeding/pumping much earlier [choice] because it was not working for me, but I was worried about not having enough formula. |
| 42 | I felt forced to continue breastfeeding even though it was physically and mentally hard on me. I constantly had to pump in addition to breastfeeding because of low milk supply. |

| | |
|---|---|
| 126 | This was awful and preventable and it is a shame that no one will ever be held accountable. Having a newborn is the most stressful period in life with feeding being in the top 2 biggest stressors. I had a baby that screamed every time I fed him for the first 8 or so weeks of his life which was mentally, emotionally and physically draining but I felt that I had to continue breastfeeding [breastfeeding challenges] because formula was not a reliable food source. My mental health was at an all time low and I'm still digging myself out of the hole that I was in during that time. |
| 1 | Ultimately the formula crisis led to me breastfeeding much longer than I had “planned” [choice] (This is my second child so I was going from past experience breastfeeding). While I know this isn’t a negative thing it created mental stress as I worried constantly about making sure I was producing enough milk during a time period when I knew I wouldn’t be able to purchase formula. |
| 141 | Mothers already have such a hard time adjusting to taking care of a baby, adding in the stress of trying to feed your baby is devastating. I personally drove [traveled] from store to store looking for formula. It was the scariest and hardest time. I was trying so hard to breastfeed but the more I stressed, the less milk [breast milk supply] I had. Formula should be free. |
| 20 | I was terrified to stop breastfeeding even though it was damaging my mental health as my milk [breast milk supply] continued to dry up despite multiple meetings and trainings with LCs. I knew when I stopped breastfeeding that I would struggle to find enough formula to feed my baby, and the stress of finding formula compounded with my postpartum depression/anxiety was very overwhelming. We are fortunate that we had family members willing to drive 2 hours away from our home to bring us a week’s worth of formula at a time. I did not feel like it was appropriate for us to stockpile, as I felt that attributed to the problem as a whole. So we had to drive [traveled] 30+ miles almost weekly to find formula for our baby. I felt like there should have been more allowance [restrictions] for other formula companies from Europe to be sold to the US- although we did use them once they were approved here. I also felt like it should have been monitored when people began stockpiling formula and that should have been stopped. I have seen so many people “reselling” all this formula they stockpiled and took it off the shelves for babies who needed it. |
| 108 | I had to spend time while at work searching where formula was in stock and then going to buy it, hoping it was there. My family and friends did the same for us. I do not think enough was done by any government agency to fix the shortage, I think it shows how women and families are really viewed by our government, we are less than men. Shelves are still not fully stocked and I view that as a huge problem, they did not fix anything just got the bare minimum back up and running. |
| 113 | In dealing with the infant formula shortage, I had to work with my in-laws in FL to ship up formula to us in Maine. As well as, my dad used his connection with retired veterans for them to send me formula as well, since I could not find any in my state.<br><br>I do feel that there should be some sort of help in financial assistance with buying formula. |

| | |
|---|---|
| 132 | I started to worry about the availability of formula before my baby was born in February because my pediatrician warned us that ready-to-feed formula was hard to get but the best thing for newborns. I bought some on Amazon but it wasn't the brand I was hoping for and this is when the anxiety started.<br><br>Breastfeeding was difficult for me and my baby so I was thankful we had ready to feed formula we got for free from the hospital in the first few days. Given the struggles we were having with breastfeeding I immediately went online to find whatever ready to feed formula I could find and bought a bunch. I didn't end up using this formula because I ultimately was able to breastfeed and didn't need the formula. I ended up giving the formula to a local friend.<br><br>The formula shortage caused me severe anxiety and stress when my baby was 3 months old because I didn't want to continue to breastfeed [choice] (because it was challenging for me and for my baby) but felt trapped because there was no other option that was readily available. I started to slowly stockpile formula in July when it started to become available on Amazon and then was able to get a subscription to Bobbie. Having enough supply of formula was the only way I felt comfortable stopping breastfeeding which I did 3 months later when he was 6 months. This was only possible because I have enough income to spend money on this effort - and I spent a lot. While I felt very guilty about stockpiling formula, as a parent I had to do what I needed to do to safely feed my baby.<br><br>The system is completely broken. The ingredients are terrible in most brands and recalls are normal. I only trust the expensive brands like Bobbie at this point which is outrageous that quality ingredients are not available to lower income families. A parent should not have to struggle to get formula to feed her baby when that might be their only food source. It is outrageous that people with less income were not able to get food for their babies and the government and private sector absolutely needs to do something to fix this broken system. Companies should be held accountable for providing safe and healthy ingredients for babies. The government should ensure there are safeguards in place for quality but also a stronger supply chain that doesn't break down so quickly and so extensively. The government should also make sure that all parents have access to formula that is needed to keep their babies alive - it should not only be accessible for the privileged. |
| 145 | I never imagined my milk wouldn’t have enough supply [low breast milk supply] to feed my baby. So I really relied on formula to keep up with his birth weight. I didn’t need as much formula as other families but I did struggle finding it to have |
| 155 | It was challenging and scary at times. It caused stress for my family and friends. |
| 199 | Make sure this doesn’t happen again. It was very scary |
| 134 | I felt like I was stuck in a hard place, mentally dealing with the struggles of breastfeeding [breastfeeding challenges] and then worrying about trying to stop but not having the formula available. |
| 60 | It's completely unacceptable that in the US in 2022 we had to experience a formula shortage. |
| 77 | none |
| 162 | The formula shortage crisis was an unnerving situation for parents across the US. Babies who are combination-fed or exclusively-formula-fed were put in jeopardy [safety]. This is unacceptable on so many levels.<br><br>Thank you for spending the time researching this issue. It is critical that the correct groups understand what went wrong and how to ensure it never happens again. |
| 19 | none |

| | |
|---|---|
| 197 | paid formula through insurance; maintaining regular checks for quality control in the factories where the formula is being made to help reduce the amount of time that factories may be shut down for an entire overhaul cleaning and rebuild to go back to the FDA standards. |
| 55 | I think having a website with all the formula brands and explaining how Formula A is the exact same formula as Formula B from this other company. I think as a first time mom what would've help me tremendously would've been a website to see what is the difference between this hundreds of different formulas. Why is one brand $17 and the other is $30. I think it would've been helpful if somehow there was a resource for families who's babies were under 6mo and exclusive formula fed<br>were reached out to to help them find the formula. I [traveled] drove to 4-6 hrs every 2 days all over Southern California to different Targets and Grocery stores. I would wake up at 4am to meet the delivery trucks at CVS or Walgreens to see if they had any of the formula I was looking for. I paid hundreds of dollars to get formula from out of state or via app that required expensive subscriptions and tips. I once paid $35 for 1 bottle of ready-feed formula bottle because the app GoPuff had it on surcharge and I had to tip the driver normally those bottles were $9.99. We're thankful we had credit cards to just add to the debt that being a first time parent brings but couldn't imagine what those other moms on my mommy groups were dealing with when WIC would not allow them to get something else and they had no cash. My husband and I mailed formula to 8 different families from the east coast because if we had it hard in the Westcoast for them it was 10x harder. This affected my post partum depression tremendously because I felt so guilty for not being to breastfeed. I felt like I had let my baby down but the stress of being a first time mom did not allow my body to produce any milk [low breast milk suupply] and I tried so hard. My baby is now 10mo and I still feel so guilty. |
| 151 | There should have been a limit that consumers can purchase from the beginning. |
| 97 | None |
| 136 | It was a challenging experience, especially for first-time parents.<br>We were on the waitlist for Bobbie formula and could not get on the list till the shortage ended. We had to ask our friends who traveled abroad to buy formula for us and received 2 Aptamils. Aptamil has sugar which we did not like it. Also, we had to switch to Enfamil which our doctor provided during the visit which made her gassy [infant issues], and she refused to drink. At some point, I did not have enough milk [low breast milk supply] and had to join the breastfeeding support calls to make sure I can express enough milk. Daycare only excepted ready formula we had to get a doctor's note for them to prep formula since there is an expiration time once provided. We started solids earlier. Overall no mother ever should fear feeding her baby! |
| 6 | None |
| 98 | My baby was born right before the shortage started and it was extremely stressful. This was my 3rd child so I already knew how to breastfeed but the logistics of a feeding a 3rd child while taking care of 2 others made it hard to do exclusively. I was fortunate to have a neighbor who oversupplied to help in the first few weeks before I was able to establish the right formula. |
| 110<br>110 | The formula shortage crisis was yet again another example of how mothers are not efficiently supported in this country. It was a scary time, where prior to giving birth I had planned on breastfeeding as long as I was able to with no pressure to continue if I had to go back to work. And then when the shortage occurred there was an anxiety about if I needed to stop breastfeeding, how difficult would it be to feed my baby. Additionally, it was scary to imagine that formula was being produced in unclean [safety] factories [manufacturers]. I would hope that going forward our government will take steps to: 1) hold domestic formula producers to the highest standards as children's lives are on the line 2) be proactive about ensuring these factories are up and running especially during events like a pandemic 3) ensure imported formula is available and American parents can make their own informed choices on what to feed their babies 4) have complete transparency about this industry so parents can plan ahead |

| | |
|---|---|
| 75 | None |
| 61 | I was wildly lucky in that I managed to get a formula subscription before the subscriptions were closed to new members, which meant I was able to access the supply of formula my baby needed. However, I did have to commit to this subscription before trying the formula with my baby, so I was incredibly anxious she wouldn't tolerate it well. Luckily it worked out, and I was able to help other moms by tracking down the formulas they needed. The whole experience was quite traumatic, though, constantly fearing that I wouldn't be able to feed my baby and thinking about the experience of those who truly couldn't. |
| 111 | It was hard, as I never planned to feed my baby the amount of formula that I needed.<br>Health authorities should implement policies that prevent a repeat, for example better policies that facilitate infant formula importation from foreign countries.<br>Better regulations to prevent what also happened with the Abbott factory as well. |
| 154 | It was stressful. I was nursing but I always had a fear that if I stopped producing milk then what will my baby drink. I bought some ready to feed bottles as backup when I saw them at the store. I gave him formula when I feel like my milk was not coming in yet. It was great that other formulas from different countries [imports] were allowed to be sold in USA due to the shortage. |
| 195 | I think more international brands should be allowed in the US [imports]. That really helped us out. We only had access to Bobbie because of a donation that we had received right before the shortage. We only switched over the kendamil for a short while and then to Bubs until 1 yr old. I don't know what we would have done if those options were not available. All the organic brand from the US did not work for my child. I hope this does not occur again here any time soon |
| 36 | The shortage was in some ways a blessing as it gave us access to imported formulas which I found were a bit "cleaner" [quality] and better tolerated by my baby. For example, we were on a partially hydrolyzed milk protein but the carb is largely corn syrup, and were able to switch to the same broken down protein but maintain lactose, which I feel is a better alternative for us |
| 205 | |
| 38 | I saw more on the ground action from local mom Facebook [social media] groups than any government operation. |
| 186 | I kept a rigorous pumping schedule and tracked my pumped milk ounces every day to try to make enough milk every day for my son. It was very stressful and challenging because I feared that if my supply dropped I wouldn't have food for my baby. When I got my menstrual cycle back 3 months poatpartum my supply dipped [low breast milk supply] during my cycle and every month thereafter during my cycle. It was difficult to keep up with 30 ounces a day pumping. I also made less milk when sick and dehydrated when I got covid, so I used up my freezer stash. I was diligent and disciplined with my pumping, but it was mentally and physically taxing. I Felt I had no other choice but to work my hardest so my baby could eat. It's a scary feeling knowing if you can't make milk and don't have formula, your bany could die. It feels helpless and scary. |
| 149 | Breastfeeding doesn't work for everyone and the fact that during the formula shortage it was pushed so hard there was little thought made for people who couldn't breastfeed due to trauma. It ended up exacerbating my PTSD and caused me to deal with worse PPD than I should have. |
| 79 | none |
| 8 | It has been extremely challenging finding formula for my son due to the shortage |
| 89 | It was scary-- many friends and families reached out to help look for formula for him, because I wasn't producing enough breast milk [low breast milk supply]. I wish the healthcare providers would've reached out to see how we are doing. |

| | |
|---|---|
| 102 | There is an instragram account @formulamom, who was a LIFE SAVER for breaking down each formula, including imported forumlas, and she doesn't give a biased opinion. simply which formula is most like another, etc. I seem to think my situation was unlike most where I had a great supply - but that ended quickly when a clogged duct turned into an abscess that needed to be surgically drained [breastfeeding challenges]. In the time of need I reached out to Bobbie customer support and shared my situation and was fortunate enough to land a spot with their subscription service before they started making it available on shelved in Target. To be honest, subscription services should be more readily available [choice]. This creates a less stressful experience and one less thing a mother needs to be concerned about when it shows up at your door every month and I think that could help with people stockpiling. Bobbie's subscription allows you to pause, change delivery date, etc without losing your spot all while not making you feel guilty for whatever your feeding journey may be. |
| 17 | I breastfed longer than I planned because of the shortage [choice]. I had an online subscription that wasn't confirmed until a few weeks before delivery but I waited until I actually received the shipment before I started weaning. It was mentally stressful to continue to pump (I exclusively pumped since week 2) longer than I had prepared to and while working full time. |
| 68 | None |
| 87 | I think future formula shortages can be avoided if it becomes easier for more companies to enter the marketplace. The infant formula market is currently dominated by just a few companies [monopoly]—when one goes down, babies everywhere feel it. Additionally, continuing to allow foreign formulas [imports] to be sold in the United States will help guard against future shortages. In my own experience, I wasn't confident in my ability to secure formula until I signed up for a subscription to a smaller/newer formula company AND knew I could find cans of foreign European formula on the shelves at my local target. |
| 78 | none |
| 169 | None |
| 73 | I had very bad PPA in fear that my baby would starve if I quit breastfeeding and pumping. I had formula shipped from all over the country to me. Then a Hurricane demolished our town and that made the shortage even worse here. Fema had expired formula available but nothing "gentle" or non expired |
| 5 | None |
| 13 | None |
| 26 | I was only spared by the brunt of it because I was following the news while pregnant and ordered a couple months worth ahead of time in case breastfeeding didn't work out with my second (it didn't with my first). I also was "grandfathered" in to a subscription with Bobbie, because they allowed ongoing subscriptions for anyone who had made a previous purchase including a two can trial pack. Without that subscription I would've been in dire straits even with my pre planning. |
| 39 | There should be a government stockpile and there should not be a monopoly with only a few providers who are held to higher safety standards to avoid major disruptions. |
| 133 | Had a very hard time finding the sensitive Enfamil formula. We finally gave up searching and went on the myorganiccompany.store where I saw the formulas they offered. At a visit to the doctor I asked about these formulas which had great reviews and were made with better ingredients. Doctor did not agree or disagree. I gave it a try and bought kendamil organic. Had it shipped to me several times until one day I found it at target and we have been using ever since. I had a very hard time breastfeeding [breastfeeding challenges] and felt horrible [guilty]thinking about ending it and there not being formula available. There was no help [helpless] from health authorities which was disappointing. I was given more information on mommy blogs. Not everyone is fortunate enough to be able to pay $30+for formula. Very stressful experience. I still do not see the enfamil sensitive formula at stores or online. |

| | |
|---|---|
| 107 | I stopped breastfeeding and pumping when I went back to work as I planned, but started pumping again for a few more months because of the formula shortage even though my baby didn’t want to breastfeed anymore [breastfeeding challenge]. He was very picky with formulas and only liked one type that we tried but they weren’t able to take on more customers, so I had to get it from someone I met online in a local baby group. It wasn’t until my baby was almost 1 that I could buy it myself and not rely on someone to buy it for us. |
| 23 | It was anxiety filled adding to an already challenging time of learning to be a parent. |
| 131 | None |
| 95 | None |
| 3 | We need more options or more ways to purchase formula from abroad [imports]. Too many restrictions considering there are so many recalls on US products. |
| 173 | Nine |
| 101 | Allow more formulas to be sold in us [monopoly] |
| 10 | none |
| 83 | Allow all European formulas to be sold in stores [imports] since the ingredients and safety procedures while manufacturing are far superior to US formula brands. Open more manufacturing plants in the US. |
| 64 | As a first time mother it was incredibly disheartening when breastfeeding did not work out for myself and my baby [breastfeeding challenges]. Then to switch to formula and have a formula crisis only made matters worse. There should have been intervention well before the crisis reached the point it did. |
| 18 | Helping people breastfeed is not a solution to the formula shortage without comprehensive paid family leave programs that gift women the time required to breastfeed successfully. |
| 137 | I was fortunate in many ways: I was able to breastfeed, my baby + I had no complications in doing so, I had the resources to pay for a lactation consultant to come to my home to get us off to a good start, and I had the money/resources to buy formula to supplement directly from online retailers (where there didn't seem to be as many stockpile restrictions vs. in-stores, which is a sad restriction for those in WIC). However, I still was a little nervous despite all of this, so I can't imagine how those moms felt who were not as lucky, needed a specialty formula, etc. etc. I also had the education and access (plus time to do research) to understand which Toddler Formula (which was readily available) met all of the Infant Needs - was just marketed differently.<br>It is a disgrace that no governing body 1. ensured the factory issues at Abbott were resolved + back into production as soon as possible, 2. made research and information more available on formula swaps (like I did w/ toddler formula), 3. gave actual action items / next steps to families instead of saying "Ask your pediatrician" (that is not sustainable!) Throughout it all, the onus was on the parents to solve. The shelves are still abysmal where I live, which is an affluent suburb outside a metropolitan city. It's shameful as a country that we allowed this to happen. (Don't get me started on the infant Tylenol shortage happening now too!) |
| 146 | None |
| 161 | No one knows the feeling of feeling helpless of not knowing if you’re gonna be able to feed your child. I would have to call multiple stores, never would they help… I would drive to other cities as there was none in mine. A lot of companies did horrible on having there stock count notified moms looking to feed baby. It was a horrible thing to happen and it could’ve been prevented. The best thing is to never happen again. I am so suprised walking into a store and seeing formula… I shouldn’t be suprised as I am. |

| | |
|---|---|
| 119 | I wanted to fully breastfeed for at least 8 months but the baby was just unable to get milk out of me so I had to exclusively pump. With my second baby, I was able to get my supply up so that we didn't have to supplement with formula but it was nearly impossible to exclusively pump while at home with two children. There was a lot of stress when my supply [low breast milk supply] and mental state started declining and I wasn't sure if I would be able to find formula. |
| 2 | I bought formula and started my twins on formula sooner than planned to help my breast milk stash last longer. I switched to a European brand formula that was easier to purchase first through direct shipping and then switched to a different brand from operation fly over. |
| 187 | |
| 85 | I have a twin with kidney disease. She cannot be on any formula. It's been incredibly difficult to find formulas safe for her. And numerous times, once we've found one that works, it's recalled. Then we have to find something else that will work for her. It's incredibly discouraging and challenging. I had to stop pumping for cancer treatment so so couldn't  breastfeed as long as i hoped to. I also have a twin on a g tube who has extreme oral version and couldn't breastfeed. |
| 21 | I was lucky enough to be able to switch to a service I subscribed to online that was much more expensive. The only reason my baby had formula is because of that service. As I stated in the survey I had a double mastectomy (previvor BRCA positive) and was unable to breastfeed. The only way my baby could eat was with formula. I felt very fortunate to be able to afford this expensive formula. Everyone should have had the same opportunity to feed their baby with what we formula is available despite price. WIC should have covered all formula, not certain ones that no longer were available but any formula the mom was able to get. |
| 121 | It was a very scary time. I had two older children at home and a baby to try and nurse/pump for and it was stressful trying to get enough milk and or get formula to supplement. I would hope something like this never happens again. I think that there need to be more rigorous safety checks on formula producers/factories and there should be protocols in place to provide backup options if a plant is compromised. |
| 178 | None |
| 41 | Need to have a plan in place to prevent from happening again |
| 56 | Lots of moms on FB and Nextdoor sharing their excess formula and donor milk. Reliance on family members to look for restocking events.<br><br>Hospitals should be able to issue stock in times of crisis to most needed babies. Free for all at the grocery store not ideal. |
| 28 | None |
| 164 | the formula we like was easy to find in April, and then became impossible to find, or the portion sizes were off and did not work for us, meaning we would have wasted a lot because it goes bad so quickly after opening. Searching for formula is stressful, I still can't find the 2 ounce bottles in any store, they had a bunch of it in April so I had no idea there was a shortage. finding the disposable preemie nipples was also incredibly difficult, so we 99% breastfed, which means I have to pump or feed every 3-4 hours. it feels like a full-time job, even in the middle of the night, I'm still pumping to keep my supply up. |
| 30 | •Had multiple family members in multiple cities (and another state) looking for the formula we were using<br>•Got on Bobbie formula waitlist<br>•Searched internet weekly for availability<br>•The FDA was AWARE of issues at the Similac factory for YEARS before this crisis occurred. The FDA is at fault for not enforcing regulations and Similac is at fault for failing to meet regulatory standards. |
| 208 | It is imperative that more than FOUR manufacturing facilities have the ability to make FDA approved infant formula [monopoly]. After the birth of a child, families should not have to worry that the food they need to feed their children is not available. |

| | |
|---|---|
| 156 | The second half of my pregnancy was during a Covid spike so I was unable to take any courses in person and online videos were not helpful. If I had known what to expect, I believe I could have been successful. I was pressured into giving my son formula early on and it affected my milk supply so I was not able to exclusively breast feed like I hoped. We changed formulas due to the shortage And had to pay for a subscription of an organic formula as that was the only thing in stock. Luckily my son had to issues to the transition. |
| 46 | None |
| 91 | None |
| 138 | I just wish I have more information on self dieting to help product more breast milk PLUS stress full. Return to work after 3 months was to have formulas ready just in case but I didn't have anything ready by then and I kept going with Breast milk and I was stressful with work and pumping. |
| 128 | none |
| 58 | None |
| 93 | None |
| 84<br>84 | I was lucky that I planned ahead to have formula on hand when the crises occurred and I needed to start supplementing my breast milk to extend the total time my baby would get at least some of my breast milk. I had to stop breast feeding to start our second round of IVF much sooner than we planned because of anti abortion legislation that was being brought to the floor for vote in my state. We wanted more children but needed IVF to have them so we had to expedite our plans which put breast feeding on the back burner. The government could keep it's nose out of my medical decisions then I could make the best decisions for my family. Regarding supplying formula, an inventory tracker would be helpful and I knew a lot of moms couldn't get the formula they needed because wic wasn't letting them switch brands. Breaking up monopolies and granting international brands FDA approval for sale in the US could help. |
| 40 | I pumped more frequently than desired after returning to work after maternity leave since it was not possible to rely on infant formula to supplement my breast milk. It was extremely inconvenient to pump as often as I did, but I wanted to ensure my baby would not be in need of food, so I extended my breastfeeding journey via pumping. At this time, I would have preferred to rely more heavily on infant formula to supplement breast milk.<br><br>We should have flown in infant formula from outside of the US much sooner than we did. I would love to see more brands/European formula available for purchase or FDA-approved in the US to give parents more options. We should not allow certain formula brands to have a monopoly on the market to the point where if a major plant shuts down we are thrown into crisis.<br><br>There should be more brands/types of breast pumps available for parents through insurance so that they can choose the best pump for their lifestyle/personal situation. In some cases, this may help extend or enable breastfeeding if desired. |
| 188 | |
| 153 | I took greater efforts to breastfeed than I would have otherwise given the stress it put on me and my family. There needs to be more support around lactation support that is free of charge both before childbirth and after for both mothers and family members. There should also be more global availability of formula brands across countries - ie. citizens of the US should not be forced to just purchase formula from the US. The government should provide subsidies for women willing to donate breastmilk to others as well as providing subsidies for women who choose to breastfeed their own babies, as it reduces the overall strain on the demand of formula. |

| | |
|---|---|
| 140 | I had to buy formula on offer up from someone in my community when I couldn't find it. It was very stressful . The government should allow more brands and keep track of supply chain better. More teaching should be done to new mothers on breast feeding. More follow up when they get home with their baby for breastfeeding teaching like home visit. Formula should be free. Not everyone has enough milk production. More education on supplementation and breastfeeding at the same time. |
| 112 | None |
| 14 | I used a Facebook group where mom's would post pictures of formula in stores so everyone knew availability. Mom's would resell formula on it. My family all had a picture of the can of formula we used and knew to buy it if they ever saw it in the store. The government needs to step in sooner. They kept saying it was not bad yet when really the crisis was at its worst. |
| 114 | I had intended to breastfeed as long as possible, but had supply issues [low breast milk supply] from early on, so we supplemented with formula as necessary, but once formula became scarce, I had to start pumping to restimulate lactation to attempt to increase my supply. It wasn't great for my mental health. Thankfully, our pediatrician encouraged us to start solids around 6 months, which took some of the pressure off, and she was approved to start mixing formula with whole milk at 9 months, which has been serving our needs thus far, even with ongoing formula shortages in our area. Overall, I had it better than some families because I was able to still breastfeed to some extent, but it highlighted exactly how useless the FDA has become in safeguarding [safety] our food supply and I believe we should open our regulations to allow for European imports. |
| 129 | It's one of the most nerve-racking and stressful situations I've ever been in. Thinking about my child not having food and my body not producing enough milk was devastating. I wish the government would put into place laws that wouldn't allow crises as such to put our babies at risk, for example, hygiene protocols at factories, people not being allowed to buy more than what is needed, and access to emergency food if signed off by a pediatrician. |
| 29 | I feel fortunate I was able to get on the bobbie.com subscription.  Before that I was scared I would have to keep switching up my 6 weeks formula,  since I could not find his formula at any stores and online. I stockpiled the formula he had at the hospital just incase, though he had not done well on it. I was scared introducing yet another formula would be even worse for an infant and his developing digestive system. Bobbie was the 4th formula in 6 weeks we had gone on. I did a trial with them a week before the height of the shortage. They were not taking new customers and had to email them to let my 6 week year old continue with their formula, I had no other choice. The unknown of my baby not getting formula or having to keep changing brands, we needed consistency.  Fortunately he did well on it and we are still on Bobbie at 9 months. This makes me emotional just typing this. |
| 90 | The US should have always allowed these brands of formula from other countries to be sold here [imports]. If they were suitable for sale during the shortage, they should be sold all the time. The only reason they weren't is because of the money hungry companies in the US. It's so scary that this was even able to happen in our country! |
| 194 | None - because this stupid country does not care. When a mother who has had a baby - especially through C-section does not get more than 6 weeks (if lucky) for maternity, the country has lost its way. Any country which does not care for mothers and pets is doomed to burn. And it burns... |
| 124 | None |

| | |
|---|---|
| 62 | My baby was just born as the infant formula shortage was happening. I was mildly concerned as my breastfeeding plan was to see how it went and roll with it - therefore my concern was that if breastfeeding did not work for us (or me), I would not have formula to feed her. My baby was 4 weeks premature and struggled to gain weight from breastfeeding. I ended up triple feeding (breastfeeding, pumping, bottle feeding) endlessly for her to gain weight [infant issues] - she only gained 1lb in her first month (5lb 11oz at birth, 5lb 2 oz the next day, 6lb 2 oz at 1 month check-up). Because of this, I began supplementing with formula combined with breastmilk to get her weight up. This turned into May 2022 when the shortage was at its height! It was already stressful to have a premature baby, a new baby period, a baby that was struggling to gain weight, and now the likelihood that I would not be able to obtain formula right as I needed it.<br><br>I had several samples of formula provided to me by the OB office and the formula companies received prior to April 2022. Those were exhausted quickly. I went to multiple stores and looked every time I went to the store to have formula as backup while I used the samples. I tried many different samples to not have to be stuck looking everywhere for missing formula.<br><br>Given the background of the shortage began with poor quality of formula at one manufacturer, the government AND the poor quality company are both at fault. I am over 10 years into working with medical devices professionally and intimately know the FDA requirements. The company failed by not having proper quality assurance protocols into place placing the undue risk onto the most vulnerable population - infants. The government failed by not catching this quality issue until infants had fallen ill due to formula. The government also failed by not having a proper supply [chain] plan to make up for shutting down one of the most major manufacturers of formula prior to shutting down the operation. There is a balance of safety (shutting down a dangerous manufacturing operation) and safety (putting infant lives at risk from food shortage). While the manufacturer should have been shut down, the FDA and other government officials should have placed full staff together to analyze the companies other formula supplies, other manufacturer supplies, and whether any part of the original manufacturer could continue producing formula because their operations were adequate OR directly oversaw manufacturing 100% quality inspection throughout the process at the manufacturers expense. |
| 109 | I think I was fortunate that I had a relatively healthy baby that didn't need specialized formula. It was scary buying formula from "unofficial" sources but, fortunately, that time period was brief. I appreciate the US government importing brands from other countries and I think maybe they could continue to do that so we don't have all of our eggs in one basket [monopoly]. Also, I do think there is just a general lack of support and education for breastfeeding / pumping mothers. There should be more information at the hospital about tongue / lip tie, what the signs are, and how that can affect breastfeeding. There should be more support for working mothers who are pumping. There should be more paid leave for mothers to be able to directly breastfeed their child. I know many working moms who had to quit giving their child breastmilk because they simply could not express enough milk while at work and away from their child. |
| 139 | It was extremely stressful and scary to not know if I was going to be able to find food for my baby. The shortage started before May and was awful for me and other parent friends. I shipped formula out of state to a friend in Tennessee whose baby needed special formula that could not be found anywhere near her. |
| 24 | I think additional access to international formulas at a subsidized or discounted rate would be beneficial in these circumstances. I was able to go online and purchase international brands but I know not all people can do that either from affordability or acces. |
| 70 | Allow more formula manufacturers in the US<br>Make EIC contract with more than just Similac<br>Have someone make a goats milk formula<br>Allow for more imports |

| | |
|---|---|
| 127 | I had to wake early in the morning before i planned to go get formula to call each Costco to see which ones had formula come in and what formula they had.<br>I also think that because infant cannot go without formula or breastmilk and is medically necessary, this should be covered by insurance. |
| 92 | Thankfully we had enough of our preferred formula in the stores around us and it lasted us longer because I was breastfeeding and my milk supply gradually increased. |
| 158 | I would have breastfed for longer if there was more free in-home support for it and the expectations about the time it takes to EBF your child was explained to me earlier in my pregnancy. I did not know how difficult breastfeeding can be and that milk does not just come in easily. It was also expensive to pay for a LC. |
| 86 | Extremely stressful having to switch my baby from formulas especially because they were I a lower growth percentile and had acid reflux. Always looking for formula or having family look. Just kept one step ahead and had to change several times to the detriment (sometimes) of my baby. |
| 189 | The infant formula shortage made it very clear that there needs to be intentional re education about infant formula and baby feeding in the US. Approved infant formula is equal to human breastmilk, and we are told a different narrative due to lactivism. This was especially apparent through families preferring to buy or use unpasteurized breast milk from family, friends, or strangers, which is incredibly dangerous. Along with the re education, it’s very obvious that the monopoly of infant formula in the US needs changed on a legislative level. I was lucky that breastfeeding went easily because too many mothers are martyrs to make it work out of being sold that it’s the only good option for feeding their baby. I was fortunate to be able to breastfeed through the worst of the shortage to only need to supplement with formula. |
| 33 | I think the infant formula shortage in this country was shameful. I consider our situation very fortunate because our family had the means to provide for our twin babies, but it is unacceptable that new families with infants had to struggle through what is already an incredibly stressful change compiled with empty store shelves and ZERO information or hope of relief. We had twin babies and I had a very difficult time pumping and even breastfeeding, so we had to supplement from the start. Our pediatrician wasn't that helpful and we paid 3X the price for liquid pre-made formula. When the British formula, Kendamil was finally available we made the switch and found it to be superior to any American made formula. We also got access to Bobbie a formula made in Vermont, which was good quality as well. Shame on US companies like Abbott labs and Similac brands for monopolizing the formula industry and because of their lack of corporate oversight, cause a shortage in the richest country in the world. I know other families suffered more than us, especially infants who needed specialty formula. |
| 200 | I did attend a breastfeeding class that did a short overview on it at my hospital during my pregnancy however it did not adequately prepare me. My baby ate fine the first time but the second on the other breast he wouldn't so we were given pumping supplies at the hospital and were told to do that and give him a bottle. That began the downfall as he wouldn't latch and we ended up giving him formula on day 3 cause he was so hungry and I wasn't able to pump very much. My milk did eventually come in but everytime we breastfed he wouldn't latch and would cry and so I pumped for about 3 weeks until I was pain and had to give up all together and go on formula. |
| 207 | Govt to support importing or ramping up local production to stabilize supply |
| 69 | None |
| 37 | None |
| 25 | I always wanted to exclusively breastfeed but I do not make enough to satisfy my baby so I’ve always had to supplement with formula. This has been a cause of anxiety. During the formula shortage, I was so anxious and pumping way more often than was good for my mental health to provide more breastmilk. Luckily, we never felt the pinch of the shortage because we had enough formula samples from the doctor and brands before the shortage but I was so nervous about how long the shortage would last. I joined a waitlist for Bobbie and researched where/what we could do if we were ever desperate for formula. The government should allow more European brands to be sold here and it should also |

| | |
|---|---|
| | subsidize formula or human milk. Mothers should not have to worry about how to feed their babies. The shortage was such a scary time and my heart broke for all the moms and babies who were most affected by it. |
| 74 | Infant formula should be free. I'm paying $208 a month on an expensive formula because it was the only one I could find during the shortage and I don't want to switch my baby's formula again. |
| 174 | Fortunately for me I had the resources to purchase formula and keep a supply during my pregnancy never realizing there was going to be a shortage. I bought it for supplementation thinking I wasn't going to be able to or want to leave the house with the pandemic going on. I never ever thought a shortage would happen. If I knew I probably would have kept expressing milk but because I wanted to work/go back to work I didn't want to deal with pumping and carrying supplies especially during the pandemic. So I stopped pumping early. This is my second baby. With my first I pumped for a year no problem but bc this was my second it was a lot busier and more work with 2 kids so I knew I had to sacrifice pumping and that time. I definitely have regrets and also mom guilt bc I stopped pumping and then a few weeks later I could t find formula. Moms on Facebook groups were so amazing and helpful. We rallied together and helped each other out with donated pumped milk or even letting each other know who has extra or what store is selling formula. I relied very little on the govt or healthcare profs bc really nobody has the kind of love and support like another mother going through the same thing. |
| 123 | I was very lucky in that I was an over-supplier of breast milk. I was able to create a massive freezer stash to feed my baby during the crisis. However, I had planned to stop pumping much earlier and prolonged my pumping experience due to the formula shortage. This led to severe mental health decline as I hated breastfeeding and pumping. So I was lucky in that I knew we had food for the baby but felt pressure to continue pumping, event at the expense of my own health, because we did not know if we could access formula. |
| 177 | None |
| 167 | None |
| 48 | I still don't understand why the shortage occured and is still occuring…I still have a difficult time finding my son's formula in stores and online. |
| 125 | From what I can tell, it didn't seem like the U.S. government was at all prepared to deal with the formula shortage (as in, it had never occurred to anyone that such a situation might occur, and if it did, what plans might need to be put in place). In my experience, parents across the U.S. came together to support one another (I'm in a new parents' group that helped one another source formula from various stores in our county) through the crisis, much moreso than relying on the government or governmental organizations. |
| 4 | The shortage was incredibly stressful, very thankful for operation fly formula. Kendamil has been an amazing formula option for our family. Breastfeeding did not go as planned and was not working we would have switched to formula sooner but there wasn't any available and breastfeeding was becoming harder and harder. |
| 192 | none |
| 81 | Access to tested, safe donor breast milk would have been hugely helpful. Also, information on which formulas are similar would have been helpful. |
| 32 | It was stressful and put more pressure on mothers to breastfeed even if they didnt want to because there was not the comfort in having access to enough formula options for your child and being able to stick with one kind. |
| 43 | This was a very stressful time for many parents who formula fed, but I am grateful that I was able to stock up on what we needed without hoarding. |

| | |
|---|---|
| 165 | none |
| 31 | I desperately wanted to exclusively nurse but it was not possible for us. My baby was in an OP position and never turned but I managed to deliver her unmedicated as I wanted to (after 3+ hrs of pushing). Immediately after delivery, while still skin-to-skin, I hemorrhaged because of a complication with my placenta. It was dangerous and scary but I was ok.<br><br>I believe it was largely as a result of these labor challenges [breastfeeding challenges] that my body never produced a sufficient [low] breast milk supply to fully feed my baby. I tried very hard, and it made me horribly anxious and unhappy during what might otherwise have been a joyous time for my family and me. The infant formula shortage added tremendous stress and guilt to my already guilt-ridden breastfeeding journey.<br><br>I visited lactation consultants (thankfully fully covered by my insurance), tried various supplements, and triple fed for 12 weeks. It was exhausting and it did not help me achieve my breastfeeding goal. At 3 months, I gave up nursing and pumped and fed formula up until 11 months. It was still exhausting! I was pumping 7 times a day and still only producing at maximum, just over half of what my baby needed.<br><br>The thought of asking birthing people to re-lactate as a way to solve future formula crisis is so ill-advised it makes me want to scream! There is already a lack of support in the workplace and in society for lactating people to nurse or pump. As a result, when people stop lactating, it is often NOT a choice. To be forced into starting to lactate all over again because our society and government cannot figure out how to make formula safe and readily available is a travesty!<br><br>In my opinion, the best way to prevent future formula shortages is to end the monopolies that a few major corporations (i.e., Abbott) have on programs like WIC, and--more broadly--to increase the number of choices and options that birthing people have when deciding how to feed their children. European formulas) [imports] that are safe and nutritious should be available in the US. Formula facilities should be held to high standards for cleanliness. The government should step in to be sure that babies are fed and that birthing people and parents have support in feeding their babies! |
| 99 | None |
| 150 | None |
| 103 | None |
| 201 | There were no international premature formulas available |

| | |
|---|---|
| 12 | 1. Lower FDA standards or accept European standards<br>2. Allow more competition in the marketplace including imported brands<br>3. Put a price ceiling on all brands - it is too costly right now<br>4. Provide free lactation support online especially after birth<br>5. Medical insurance should cover the cost entirely and allow subscription service to your door to cut down on hoarding or buying with fear of running out<br>6. Hospitals should educate at birth about formula and donor milk - perhaps with literature moms can read after delivery. It should also include info on how moms can donate. Formula/donor milk used to be shunned at hospitals and it should be offered upfront<br>7. Medical insurance should also cover exclusively breastfeeding moms to get their breast milk tested regularly to make sure it contains appropriate nutrients and also cover cost of extra mom supplements like DHA, vitamin D and others<br>8. Disallow brand exclusions for those on WIC<br>9. Allow easier US-based formula startups with subsidies and other incentives<br><br>My baby was born March 3 and when the nurse at the hospital told me hospitals are having trouble getting formula, I went back to my hospital bed and ordered the last 4 available boxes of formula I saw online at Target. After a few weeks of supplementing formula and breastfeeding, I stopped feeding formula though I had some inventory because I feared losing my milk supply. I preferred strongly to supplement with formula but my baby stopped accepting it when I tried to reintroduce it (once stock was available and I thought it was reliably there). So now I am exclusively breastfeeding without even bottles which my baby also rejected. It has been a difficult road but not the worst as it has been for some others. It is a shame we had this crisis in America. |
| 163 | Breast feeding was not an option so I pumped. Pumping triggered major PPD for me and caused me to go on medications. Since I didn't have confidence on accessing formula, I went longer than planned |
| 66 | None |
| 82 | Allow more imported brands into the US |
| 49 | |
| 142 | Thankfully we had kind nurses in the NICU who were willing to give us some formula to us during the shortage, so we were able to manage.<br><br>However, it's unbelievable in a developed country like the US one could possibly face a situation where there would be no formula to feed their babies, particularly for mothers who have to work due to lack of paid maternity leave because of which they are not able to breastfeed or pump sufficient quantities of breast milk. |
| 176 | We had the luxury of being able to afford formula that we purchased online from overseas suppliers (and the associated shipping costs)... many mothers/families did not have that option available. Without that, I am not sure what we would have done. |
| 122 | |
| 166 | None |
| 27 | I think it was a supply chain issue or too many containers were being held at the border. Wish they'd feed our baby's first. So regulating what is held at the border. |
| 120 | I feel like I was lucky and was able to find formula when I needed it. I was nervous at first because he was on a different formula for sensitivity, but our pediatrician said he could probably tolerate different ones better than before. I think that there should always be a plan in place now to |

| | |
|---|---|
| | watch the stock of formula and to trigger some type of assistance or program when stock is getting low. Some women like me don't want to/can't re-lactate and we need formula to be stocked. |
| 115 | None |
| 106 | The stress about where to find the next jar of formula was the worst part, and while your survey states that the crisis peaked in May 2022, it was already a major concern in March 2022 when I had my baby. I had exclusively formula fed my first child in 2021 and had plans to do this again, but instead felt forced to breastfeed this March 2022 baby due to the uncertainty of the formula supply. I'm thankful that I was able to do so, at the sacrifice of my overall comfort and mental well-being, because I'm not sure that I would have been able to buy enough formula to feed him if that was his only food supply. I spent hours each day scouring the internet and store apps finding formula for myself and friends, and this mental toll was exhausting. |
| 59 | I was able to buy a can here and there but I was so afraid of breastfeeding not working out or stopping breastfeeding because I didn't know how I would feed my child. |
| 160 | There should be more formula manufacturers in the United States. |
| 105 | I wished to stop breastfeeding when my baby turned 6 months but because of the formula shortage, I could not stop until she was 11 months old. As my milk supply greatly decreased over time, I experienced increasing anxiety and stress over how I would feed my baby… which continued to impact my supply, too. |
| 34 | Not all moms can exclusively breastfeed. Not all moms have work situations that will allow pumping (despite the law). I knew all of this from the first child. I had to rent a pump from the hospital at $90/month to get more milk this time around. I had wanted to breastfeed/pump for longer but just couldn't manage it.<br>I started supplementing with the Whole Foods formula. Around 14 weeks, I noticed it wasn't on the shelves. No one at Whole Foods or Amazon could tell me anything. I switched to Burt's Bees. Transitioning was a mess. My baby cried all day and night for three days. I was back at work and had to hire help at night to manage this so that I could sleep. My older child had to switch formulas for different reasons in 2018/19 so I knew what to anticipate.<br>Then Burt's Bees stopped making formula around five months. I switched to Happy Baby Organics. I had to hire help again to deal with the change so that I could work. Now this formula is out of stock for the 6-12 month formula that I need. I may see if I can get it on Instacart even if I have to overpay. When I switched, Happy Baby had assured me that this wouldn't happen.<br>I live two minutes from Target. They sell Kendamil Organic. That'll be my next move if I can't get enough Happy Baby for the next few months. I'll have to restart my maternity leave when we switch. Luckily, I have two weeks paid time left to take before the baby turns one.<br>Even if I have gone way over budget on all of the above and have had to sacrifice in other ways, I'm lucky to have been able to do this. I'll never forget in May 2022 looking online and in pharmacies and seeing absolutely nothing available. I won't import from third party sellers because I have safety concerns. It was all terrifying. Thank you for making sure this won't happen again. |
| 170 | There needs to be more competition in the US manufacturing market for infant formula. |
| 67 | None |

| | |
|---|---|
| 171 | I planned to exclusively breastfeed for about six months, then combo feed thru 1 year. I wasn’t producing enough early enough so had to introduce formula for my daughter to make weight check ins in the first month. The fact that we had to pivot our mindset and add a new form of feeding so soon after birth, let alone during a formula shortage, increased my anxiety exponentially and likely contributed to some postpartum depression. We were lucky enough to find what we needed and afford the price of a large order. I can’t imagine how much worse my depression would have been if we had not been able to afford it.<br><br>I still watch the formula amounts like a hawk and hate wasting any of it, likely because I know how critical every ounce had been to get. It’s not something I take for granted. |
| 76 | None |
| 152 | Allow international brands to be sold in US. I found that my baby tolerated this brand better, it doesn't smell like US formula, doesn't stain clothing, seems very much to be superior to US brands. Also, formula costs are way too high, with more companies in the business prices would be able to come down. Breastfeeding is very difficult and still not socially acceptable. There need to be way more breastfeeding accommodations being made to make it a more realistic option for families (i.e. mothers rooms with sinks, outlets, etc. |
| 100 | More formula companies should be able to be created. More support for women breast feeding and pumping to learn how to help baby latch and pump successfully. I am pumping almost 12 months and still giving random formula when I don’t make enough. Pumping is hard and has a lot of little tricks to make it easier, I had to find women on social media to guide me. There is no support at the hospital or with lactation consultants, just ‘here’s the pump’.<br><br>Still, when I go to the store, the formula I want to use is out, even now almost a year later. I noticed the shortage back in December of 2021, I wanted to have some bottles just incase, because my first was mostly formula feed, I couldn’t find the formula I used with him (Similac Pro-Advance) and this was way before the officially formula shortage. In my opinion there was a massive cover up by the government on this shortage, to protect the formula companies. Anyone should be able to make formula as long as they go through the proper checks and balances to make it safe.<br><br>Thank you for doing this research, I hope we can make some change in this space so mothers and fathers and babies don’t go through what we did this year. |
| 198 | I probably would have supplemented with formula more earlier than I did if there was not a shortage. I was so scared to introduce formula incase we could not find it and my baby only preferred formula, so I breastfed longer than I wanted to. It took a toll on my mental health. |
| 57 | The formula we used was only available online or at target. It made it hard to only have one place to be able ti go to look for the formula when everyone was trying to get the same thing. |
| 71 | None |
| 148 | It is scary not knowing if you will be able to feed your baby. I was pumping but could not keep up with my baby's demands and needed to supplement with formula. I would say it was ridiculous that people were able to hoard formulas during that time. It would have been very helpful if stores would have limited the amount people could buy at once. |
| 157 | Should never happen and the government should be able to assist any parent in need of feeding their infant. |
| 213 | |
| 144 | It was unbelievably stressful and every time we purchase a pack of formula we were so lucky. We never bought more than 1 pack, knowing another family would be in need. |

| | |
|---|---|
| 63 | It was a very stressful time. I didn't produce enough milk and my baby was losing weight. I was very concerned about the type of formula I fed her and didn't want to use the typical American formulas. I used every resource I could to help find her the formula she needed. The shortage picked up right when I started exclusively using formula. I bought formula off of random people (picked up from their homes), coordinated to arrive at stores right after the delivery arrived, had people ship me the formula we needed, etc... |
| 35 | None |
| 16 | None |
| 9 | None |
| 47 | I think the monopoly that exists in the united states is horrendous, not to mention what we think is healthy formula is full of crap products. The formulas produced in Europe, New Zealand, Australia, etc. are much cleaner ingredients and I wish we didn't need our formula to go through such regulatory processess. |
| 143 | I had a uti and stopped breastfeeding because I had to take antibiotics. Then I started again, but not much was coming out. That’s when I started formula. The baby was only able to really tolerate nutramegen. When the shortage happened I struggled to get it. Then I switched to bobbie and byheart. I picked them because it was shipped to my house and I’d never run out. My baby didn’t do well on it. I wish I was able to breastfeed and there were more resources available to assist. I ended up with a European formula that target started selling. The baby has since tolerated this one. |
| 94 | I was scared I wouldn’t get formula for my baby because I was not producing enough breastmilk and was also nervious about going back to work and having to deal with pumping while at work. Fortunately, our good friends in LA have been subscribed to Bobbie since their baby was born in 2021 and help us use their subscription to get formula for our baby. We were stress free since May 6th 2022 about providing formula for our baby because of our friends help to use their Bobbie subscription. |
| 65 | While I recognize I was fortunate to find a friend who was able to sell me her monthly subscription of Bobbie formula after she weaned her baby, I spent a few weeks panic-buying anything I could find. I'm also picky about ingredients and really wanted to have him on Bobbie. No one should have to experience the stress we experienced during those months! I was returning to work at that time and it was an added stressor for sure. |
| 11 | None |
| 183 | We were lucky that I could primarily pump and breast feed but I was afraid of how I would be able to feed him if my supply dropped. It was very stressful. |
