# Supplementary material for: Cross-Sectional Analysis of Infant Diet, Outcomes, Consumer Behavior and Parental Perspectives to Optimize Infant Feeding in Response to the 2022 U.S. Infant Formula Shortage
Source: Nutrients. 2024 Mar 5;16(5):748. doi: 10.3390/nu16050748 (PMC10934383; doi:10.3390/nu16050748)
Supplement: Supplementary file 1 [file nutrients-16-00748-s001.zip › Supplementary FileS1_Survey_231223.pdf]

UC DAVIS
UNIVERSITY OF CALIFORNIA

Questionnaire Instructions

Infant Formula Shortage Crisis Survey

Thank you for taking the time to complete this survey!

Your participation is voluntary.

This survey will take you approximately **15 to 30 minutes** to complete.

This survey will ask you questions about your baby's birth and delivery, diet, and your experience during the infant formula shortage that occurred around April through June 2022 and peaked in **May 2022**.

We hope to use this information to create resources that could help prevent another infant feeding crisis.

You are being asked to complete this survey because you meet all six conditions:

1) **You** are **18 years or older**.

2) You **and** your baby lived in the **United States** in May 2022.

3) You are a parent of a **baby** (biological or not biological) who was **6 months old or younger** in May 2022.

4) Your **baby** consumed **some amount** of infant formula before the May 2022 shortage.

5) You **experienced challenges** with feeding your baby because of the infant formula shortage in May 2022.

6) You **agree** that only one parent of one baby from the same household will complete the survey. If you have multiple babies (for example, twins, triplets, etc.), only one survey may be submitted for one of the babies.

Compensation

The first 100 people who complete the survey will receive an electronic $50 Target gift card by email.

Gift card recipients will be contacted in February 2023. You will be asked to confirm your email address.

In order to receive a gift card, only one parent of one baby from the same household may complete this survey.

Survey Instructions

You may stop taking the survey and re-open it at the last spot you left off on any device using the link we sent to your email address. Once you begin the survey, you will have **2 weeks** to complete it.

Please answer the questions honestly and to the best of your knowledge; you will *not* be able to go back and edit your responses to some parts of the survey.

Your answers will be kept confidential.

Once you submit your completed survey, you will not be able to go back and change your answers.

The survey closes on **January 31, 2023**.

For any questions or concerns, please email the Foods for Health Team at UC Davis at foodsforhealth.research@gmail.com.

Survey criteria to match survey 1

What is **your** name?

First name: ____

Last name: ____

What is your **baby's** name?

First name: ____

Last name: ____

Did your baby consume any amount of infant formula ***before*** the **infant formula shortage** that peaked in **May 2022**?

- Yes
- No
- Unsure
- Decline to answer

Did your baby consume any amount of infant formula ***during*** the **infant formula shortage** that peaked in **May 2022**?

- Yes
- No
- Unsure
- Decline to answer

Demographics

Demographics

In which state do you currently reside?

What is your U.S. zip code

Zip code:

What is your **ethnicity**?

- Hispanic or Latinx
- Not Hispanic or Latinx
- Unsure
- Decline to answer

Which **race** best describes you?

- American Indian/Alaska Native
- Asian
- Native Hawaiian or other Pacific Islander
- Black or African American
- White (including Middle Eastern populations)
- Other (please describe)
- 2 or more (please describe)
- Unsure
- Decline to answer

What is the highest level of **education** you have completed?

What is your current **marital status**?

- Married/Unmarried couple
- Divorced/Separated
- Widowed

- Never married
- Decline to answer

How **many individuals** live in your household?

[A household includes one or several persons who live in the same home and share meals. Include yourself plus any other people who live in your household part- or full-time.]

What was your **combined household income** before taxes in the past year?

[Please include your income plus the income of all of the people in your household from all sources such as wages, salaries, Social Security or retirement benefits, help from relatives, etc.]

Have **you or your baby's co-parent** received any **benefits** from the Special Supplemental Nutrition Program for Women, Infants, and Children otherwise known as **WIC** within the **past 12 months**?

- Yes
- No
- Unsure
- Decline to answer

What was **your** assigned sex at birth?

[We are asking about your sex to follow-up with questions about your reproductive history.]

- Female
- Male
- Intersex
- Decline to answer

Questions about your reproductive history

How many times have you **ever been pregnant**?

*[Count **all** pregnancies including those that ended in live birth, stillbirth, miscarriage, abortion or a tubal, ectopic, or molar pregnancy. Include pregnancies from other relationships.]*

How many times have you **delivered a live birth**?

[Include children from other relationships.]

Questions about baby & relationship to baby

Questions about your baby

Do you have a baby that was born between November 2021 and May 2022?

- Yes
- No
- Unsure
- Decline to answer

How did this baby join your family?

[For this question we are only asking about your baby that was born between November 2021 and May 2022.]

- I was pregnant with and delivered this baby
- My partner was pregnant with and delivered this baby
- This baby was born by surrogacy
- This baby was adopted

- Other
- Decline to answer

How many babies were born at the same time?

[For this question we are asking about the number of babies that were delivered by the same person between November 2021 and May 2022. Examples include a single baby or multiple birth such as twins, triplets, etc. Include all babies that are living or were born by stillbirth.]

- Singleton (a single baby)
- Twins (two babies)
- Triplets (three babies)
- Quadruplets (four babies)
- More than four babies

How many of these babies currently live with you?

- 1
- 2
- 3
- 4
- More than 4

What is your **baby's date of birth**?

[For multiple births, list the date of birth of your oldest baby.]

	Month	Day	Year

What was the **gestational age** of your baby **at birth**?

[For this question we are asking if your baby was born at term which is 37 weeks or later or was born premature which is before 37 weeks. For multiple births, use the gestational age of your oldest baby.]

- Term (born 37 weeks or after)
- Preterm (born before 37 weeks)

- Unsure
- Decline to answer

What was the **gestational age** of your baby **at birth** in <u>weeks and days</u>?

[If you are unsure of your baby's gestational age at birth, please estimate to the best of your abilities. For multiple births, use the gestational age of your oldest baby.]

	Weeks	Days

Infant Feeding History

Questions about feeding your baby

Did you <u>ever</u> breastfeed or feed your expressed milk to your baby?

- Yes
- No
- Unsure
- Decline to answer

At what age did your baby **first** drink <u>any amount</u> of infant formula?

[Please estimate if you are unsure. Answer in weeks OR months only. If your baby was younger than 1 week, please write "1" in the "weeks" box. If you have multiple babies (for example, twins or triplets, etc.) you may answer the same response for all of these babies.]

Weeks:

Months:

Questions about feeding your baby ***before*** the May 2022 infant formula shortage

What did your baby **typically** eat over ***7 days right before*** the **May 2022 infant formula shortage**?

[check all that apply]

Human milk

- ☐ Breast milk (baby's mother's breast milk)
- ☐ Breast milk from a surrogate who was pregnant with my baby
- ☐ Informal or casual breast milk sharing (someone else's breast milk that is not from a milk bank)
- ☐ Pasteurized donor human milk from a milk bank

Formula

- ☐ Infant formula
- ☐ Expired infant formula
- ☐ Homemade infant formula
- ☐ Watered-down infant formula
- ☐ Premature formula
- ☐ Toddler formula

Other Milks

- ☐ Cow milk
- ☐ Evaporative cow milk powder
- ☐ Watered-down cow milk
- ☐ Goat milk
- ☐ Watered-down goat milk
- ☐ Plant-based milks (coconut, almond, oat, soy, rice, cashew, etc.)

Other Foods and Drinks

- ☐ Baby cereal
- ☐ Eggs
- ☐ Fish or shellfish (including purees)
- ☐ Fruit (including purees)
- ☐ Meats, chicken, combination dinners (including purees)
- ☐ Nut butters or other nut containing foods
- ☐ Other cereals and starches: breakfast cereals, teething biscuits, crackers, breads, pasta, etc.
- ☐ Other dairy foods: yogurt, cheese, ice cream, pudding, etc.
- ☐ Other soy foods: tofu, frozen soy desserts, etc.
- ☐ Sweet drinks: juice drinks, soft drinks, soda, sweet tea, Kool Aid, etc.
- ☐ Sweet foods: candy, cookies, cake, etc.
- ☐ Vegetables (including purees)

- ☐ 100% fruit or 100% vegetable juice
- ☐ Other (please describe) ______

How sure are you about what your baby ate over ***7 days right before*** the **May 2022 infant formula shortage**?

- ○ Not at all sure
- ○ Slightly sure
- ○ Somewhat sure
- ○ Moderately sure
- ○ Extremely sure

How did you obtain informal shared breast milk?

[check all that apply]

- ☐ I **bought** it from a friend or family member
- ☐ I **bought** it from someone I did not know through the internet or social media
- ☐ I received it for **free** from a friend or family member
- ☐ I received it for **free** from someone I did not know through the internet or social media
- ☐ Other (please describe) ______

What were the infant formula **brands** your baby **typically** ate over 7 days right before the **May 2022 infant formula shortage**?

[check all that apply]

- ☐ U.S. brands
- ☐ Imported or international brands
- ☐ Unsure

How did you obtain infant formula ***before*** the **May 2022 infant formula shortage**?

[check all that apply]

- ☐ I **bought** formula from stores (including grocery stores, pharmacies, etc.)
- ☐ I **bought** formula online from infant formula websites

- ☐ I **bought** formula online from 3rd party websites (eBay, Craigslist, Amazon, myorganiccompany.store, etc.)
- ☐ I **bought** formula through social media (Facebook, TikTok, Instagram, Twitter, etc.)
- ☐ I received **free** formula from my baby's healthcare provider
- ☐ I received **free** formula through social media (Facebook, TikTok, Instagram, Twitter, etc.)
- ☐ Formula was **shipped** to me from friends/family from **within the U.S.** (including both free or purchased formula)
- ☐ Formula was **shipped** from friends/family from **overseas** (including both free or purchased formula)
- ☐ Other (please describe) ____________
- ☐ Unsure

What online 3rd party websites did you use to purchase infant formula?

[Write "unsure" if you do not remember.]

____

Did your baby require a **specialty infant formula** during the **infant formula shortage** due to a medical or metabolic condition?

[Specialty infant formulas are designed to give your baby certain nutrients and assist with certain health or feeding issues. Specialty infant formulas may be recommended by a doctor for food allergies, sensitivities, gastrointestinal concerns, malabsorption, infants born premature, etc.]

- ○ Yes
- ○ No
- ○ Unsure
- ○ Decline to answer

Why did your baby require a **specialty infant formula**?

[check all that apply]

- ☐ Allergy (including cow milk protein allergy)
- ☐ Colic
- ☐ Constipation
- ☐ Gas
- ☐ Intolerance
- ☐ Malabsorption

- ☐ Metabolic condition (including galactosemia, phenylketonuria, etc.)
- ☐ Prematurity
- ☐ Rashes
- ☐ Reflux
- ☐ Other (please describe) ____________
- ☐ Decline to answer

Which **type** of infant formula did you use ***before*** the **May 2022 infant formula shortage**?

[check all that apply]

- ☐ Extensively hydrolyzed (examples: Enfamil Nutramigen, Enfamil Pregestimil, Similac Alimentum, or Perrigo Store Brand HA)
- ☐ Free amino acid based (examples: Neocate, EleCare, PurAmino, or Alfamino)
- ☐ Goat milk (example: Kabrita)
- ☐ Intact Cow's milk protein (including added rice formulas) (examples: Similac Advance, Pro-Advance, Spit-upor Enfamil NeuroPro, AR, or Bobbie)
- ☐ Partially hydrolyzed/Gentle (examples: Enfamil NeuroPro Gentlease, Gerber Good Start Gentle or Similac Pro-Total Comfort)
- ☐ Premature infant formula (examples: Enfamil NeuroPro Enfacare, Enfamil premature infant formula 24 cal high protein, or Similac Neosure)
- ☐ Sensitive/Reduced lactose (examples: Similac or Enfamil Sensitive)
- ☐ Soy protein (examples: Prosobee or Isomil)
- ☐ Other (please describe) ____________
- ☐ Unsure

Did you **switch** infant formula(s) from one brand or type to another ***during*** the **May 2022 infant formula shortage**?

- ○ Yes
- ○ No
- ○ Unsure
- ○ Decline to answer

Did you switch formula(s) because you could not find the infant formula(s) you typically used?

[For this question we are asking if you had to switch formula(s) because you could not purchase it anywhere, including in stores or online.]

- Yes
- No
- Unsure
- Decline to answer

Approximately, **how many times** have you had to switch the infant formula(s) you fed to your baby ***during*** the **May 2022 infant formula shortage**?

- 1-2
- 3-5
- More than 5
- Unsure

Which **type** of infant formula did you **switch** to ***during*** the **May 2022 infant formula shortage**?

[check all that apply]

- ☐ Extensively hydrolyzed (examples: Enfamil Nutramigen, Enfamil Pregestimil, Similac Alimentum, or Perrigo Store Brand HA)
- ☐ Free amino acid based (examples: Neocate, EleCare, PurAmino, or Alfamino)
- ☐ Goat milk (example: Kabrita)
- ☐ Intact Cow's milk protein (including added rice formulas) (examples: Similac Advance, Pro-Advance, Spit-upor Enfamil NeuroPro, AR, or Bobbie)
- ☐ Partially hydrolyzed/Gentle (examples: Enfamil NeuroPro Gentlease, Gerber Good Start Gentle or Similac Pro-Total Comfort)
- ☐ Premature infant formula (examples: Enfamil NeuroPro Enfacare, Enfamil premature infant formula 24 cal high protein, or Similac Neosure)
- ☐ Sensitive/Reduced lactose (examples: Similac or Enfamil Sensitive)
- ☐ Soy protein (examples: Prosobee or Isomil)
- ☐ Other (please describe) ______
- ☐ Unsure
- ☐ I did not switch the type of formula I fed my baby

Did your baby have any of the following problems with the new infant formula you switched to ***during*** the **May 2022 infant formula shortage**?

[check all that apply]

- ☐ An allergic reaction or food intolerance
- ☐ Constipation
- ☐ Diarrhea
- ☐ Eczema (atopic dermatitis)
- ☐ Fever
- ☐ Fussy or irritable
- ☐ Too much mucus
- ☐ Too much gas
- ☐ Too much spit up
- ☐ Vomiting
- ☐ Other (please describe) __________
- ☐ None of the above
- ☐ Unsure

Questions about feeding your baby ***during*** the May 2022 infant formula shortage

---

What did your baby **typically** eat over ***7 days during*** the **May 2022 infant formula shortage**?

[check all that apply]

Human milk

- ☐ Breast milk (baby's mother's breast milk)
- ☐ Breast milk from a surrogate who was pregnant with my baby
- ☐ Informal or casual breast milk sharing (someone else's breast milk that is not from a milk bank)
- ☐ Pasteurized donor human milk from a milk bank

Formula

- ☐ Infant formula
- ☐ Expired infant formula
- ☐ Homemade infant formula
- ☐ Watered-down infant formula
- ☐ Premature formula
- ☐ Toddler formula

Other Milks

- ☐ Cow milk
- ☐ Evaporative cow milk powder
- ☐ Watered-down cow milk
- ☐ Goat milk
- ☐ Watered-down goat milk
- ☐ Plant-based milks (coconut, almond, oat, soy, rice, cashew, etc.)

Other Foods and Drinks

- ☐ Baby cereal
- ☐ Eggs
- ☐ Fish or shellfish (including purees)
- ☐ Fruit (including purees)
- ☐ Meats, chicken, combination dinners (including purees)
- ☐ Nut butters or other nut containing foods
- ☐ Other cereals and starches: breakfast cereals, teething biscuits, crackers, breads, pasta, etc.
- ☐ Other dairy foods: yogurt, cheese, ice cream, pudding, etc.
- ☐ Other soy foods: tofu, frozen soy desserts, etc.
- ☐ Sweet drinks: juice drinks, soft drinks, soda, sweet tea, Kool Aid, etc.
- ☐ Sweet foods: candy, cookies, cake, etc.
- ☐ Vegetables (including purees)
- ☐ 100% fruit or 100% vegetable juice

☐ Other (please describe) ____________

How sure are you about what your baby ate over ***7 days during*** the **May 2022 infant formula shortage**?

- ○ Not at all sure
- ○ Slightly sure
- ○ Somewhat sure
- ○ Moderately sure
- ○ Extremely sure

How did you obtain informal shared breast milk?

[check all that apply]

- ☐ I **bought** it from a friend or family member
- ☐ I **bought** it from someone I did not know through the internet or social media

- [ ] I received it for **free** from a friend or family member
- [ ] I received it for **free** from someone I did not know through the internet or social media
- [ ] Other (please describe) ______

What were the infant formula **brands** your baby **typically** ate over 7 days during the **May 2022 infant formula shortage**?

[check all that apply]

- [ ] U.S. brands
- [ ] Imported or international brands
- [ ] Unsure

How did you obtain infant formula ***during*** the **May 2022 infant formula shortage**?

[check all that apply]

- [ ] I **bought** formula from stores (including grocery stores, pharmacies, etc.)
- [ ] I **bought** formula online from infant formula websites
- [ ] I **bought** formula online from 3rd party websites (eBay, Craigslist, Amazon, myorganiccompany.store, etc.)
- [ ] I **bought** formula through social media (Facebook, TikTok, Instagram, Twitter, etc.)
- [ ] I received **free** formula from my baby's healthcare provider
- [ ] I received **free** formula through social media (Facebook, TikTok, Instagram, Twitter, etc.)
- [ ] Formula was **shipped** to me from friends/family from **within the U.S.** (including both free or purchased formula)
- [ ] Formula was **shipped** from friends/family from **overseas** (including both free or purchased formula)
- [ ] Other (please describe) ______
- [ ] Unsure

What online 3rd party websites did you use to purchase infant formula?

[Write "unsure" if you do not remember.]

______

Questions about your experiences and opinions related to the infant formula shortage

Who has provided you with **guidance or support** to help you feed your baby during the **May 2022 infant formula shortage**?

[check all that apply]

- ☐ Birthing or baby care class
- ☐ Blogs
- ☐ Breastfeeding support group
- ☐ Doctor, physician assistant, or nurse practitioner
- ☐ Doula
- ☐ Lactation consultant or lactation counselor
- ☐ News or magazines (including TV broadcast, online articles, etc.)
- ☐ Nurse or midwife
- ☐ Nutritionist or dietitian
- ☐ Radio or podcasts
- ☐ Relatives or friends
- ☐ Social media (Facebook, Instagram, Twitter, etc.)
- ☐ Telephone support helpline or hotline
- ☐ Videos (YouTube, documentaries, etc.)
- ☐ Websites by health authorities (CDC, healthcare organizations, etc.)
- ☐ Websites by infant formula companies
- ☐ Websites by other groups
- ☐ Women, Infants, Children (WIC) food program
- ☐ Other (please describe) ____________
- ☐ None of the above

Rate how **helpful** the following resources **have been** with providing you with **guidance** or **support** to help you feed your baby during the **May 2022 infant formula shortage**.

[For this question, move the marker to the number that best represents how helpful each resource was to you. 0 equals not helpful at all, 10 equals most helpful and 5 is somewhere in the middle.]

Not helpful at all — Most helpful

0 1 2 3 4 5 6 7 8 9 10

Resource	Value
» Birthing or baby care class	6
» Blogs	0
» Breastfeeding support group	0
» Doctor, physician assistant, or nurse practitioner	0
» Doula	0
» Lactation consultant or lactation counselor	0
» News or magazines (including TV broadcast, online articles, etc.)	0
» Nurse or midwife	0
» Nutritionist or dietitian	0
» Radio or podcasts	0
» Relatives or friends	0
» Social media (Facebook, Instagram, Twitter, etc.)	0
» Telephone support helpline or hotline	0
» Videos (YouTube, documentaries, etc.)	0
» Websites by health authorities (CDC, healthcare organizations, etc.)	0
» Websites by infant formula companies	0
» Websites by other groups	0
» Women, Infants, Children (WIC) food program	0
» Other (please describe)	0

	Not helpful at all										Most helpful
	0	1	2	3	4	5	6	7	8	9	10

${q://QID156/ChoiceTextE

» None of the above 0

How did your baby's healthcare provider **guide** or **support** you during the **May 2022 infant formula shortage**?

[check all that apply]

- ☐ Recommended stores where I could purchase infant formula
- ☐ Recommended websites where I could purchase infant formula
- ☐ Recommended switching from one formula to another
- ☐ Recommended switching to a toddler formula
- ☐ Provided coupons
- ☐ Provided samples of infant formula
- ☐ Provided information about lactation and breastfeeding
- ☐ Provided information about relactation (restarting breastfeeding)
- ☐ Provided educational materials or referrals to websites with information about what to do during the infant formula shortage
- ☐ Provided resources to obtain donor human milk
- ☐ None of the above

How **many** infant formula samples did you receive from a healthcare provider?

[Infant formula samples include ready-to-feed bottles and full-sized cans of infant formula.]

- ○ 1 to 2
- ○ 3 to 4
- ○ 4 to 5
- ○ 5 to 6
- ○ More than 6

How **helpful** do you think the following activities **would be** to help families feed their babies in the near future.

[For this question, move the marker to the number that best represents how helpful each activity could be in helping families feed their babies. 0 equals not helpful at all, 10 equals most helpful and 5 is somewhere in the middle. Select "Unsure" for activities you are unsure about.]

	Not helpful at all									Most helpful	Unsure
	0	1	2	3	4	5	6	7	8	9	10	
Expectant mothers receive free lactation education during pregnancy to help prepare them to confidently breastfeed their babies												☐

	0	1	2	3	4	5	6	7	8	9	10	Unsure
Mothers receive free lactation support from lactation consultants after their babies are born												☐

	0	1	2	3	4	5	6	7	8	9	10	Unsure
Health insurance and Medicare pay for pasteurized donor milk												☐

	0	1	2	3	4	5	6	7	8	9	10	Unsure
Pasteurized donor milk is sold at discounted rates												☐

	0	1	2	3	4	5	6	7	8	9	10	Unsure
Pasteurized donor milk is free												☐

	Not helpful at all									Most helpful		Unsure
	0	1	2	3	4	5	6	7	8	9	10	
Health insurance and Medicare pay for **imported**, commercially-available infant formulas												☐

	0	1	2	3	4	5	6	7	8	9	10	Unsure
Health insurance and Medicare pay for all **U.S.**, commercially-available infant formula brands												☐

	0	1	2	3	4	5	6	7	8	9	10	Unsure
Parents can choose to use any commercially-available infant formula brand without restrictions by health insurance or Medicare												☐

	0	1	2	3	4	5	6	7	8	9	10	Unsure
Measures are created to prevent customers from stockpiling infant formula from stores												☐

	0	1	2	3	4	5	6	7	8	9	10	Unsure
Mothers receive free lactation support from lactation consultants to help them relactate (restart breastfeeding)												☐

	Not helpful at all									Most helpful		Unsure
	0	1	2	3	4	5	6	7	8	9	10	
Online videos that help explain how to relactate are freely accessible (restart breastfeeding)												☐

	0	1	2	3	4	5	6	7	8	9	10	Unsure
Information that lists where infant formula can be purchased in stores is available online												☐

	0	1	2	3	4	5	6	7	8	9	10	Unsure
Information that describes which infant formula brands are similar and meet each baby's unique health needs is available online												☐

	0	1	2	3	4	5	6	7	8	9	10	Unsure
Recipes on making homemade infant formula designed by infant nutrition experts are available online												☐

	Not helpful at all									Most helpful		Unsure
	0	1	2	3	4	5	6	7	8	9	10	
Parents receive free bottle feeding education during pregnancy to help prepare families to confidently feed their newborn												☐

	0	1	2	3	4	5	6	7	8	9	10	Unsure
More infant formula brands are available through WIC												☐

	0	1	2	3	4	5	6	7	8	9	10	Unsure
More infant formula brands are allowed to produce and sell infant formula in the United States												☐

Please select the answer that best describes your opinion.

	Strongly disagree	Disagree	Neither agree or disagree	Agree	Strongly agree
Ready-to-feed infant formula is safe for babies	○	○	○	○	○
Concentrated liquid infant formula is safe for babies	○	○	○	○	○
Infant formula powder is safe for babies	○	○	○	○	○

Please select the answer that best describes how you relate to each of following situations related to the infant formula shortage.

	Strongly disagree	Disagree	Neither agree or disagree	Agree	Strongly agree
I didn't have enough food for my baby	○	○	○	○	○
I was concerned about what I would feed my child if I stopped breastfeeding	○	○	○	○	○
I was concerned about how my baby would tolerate a new infant formula that was available	○	○	○	○	○
I fed my baby solid food earlier than I had originally planned	○	○	○	○	○

What is the **most amount** of infant formula you had at home during the **May 2022 infant formula shortage**?

[For this question, think about the amount of infant formula as the number of days or weeks you could feed it to your baby.]

- ○ 1 to 4 days
- ○ 5 to 9 days
- ○ 10 days to 2 weeks
- ○ 2 to 4 weeks
- ○ 4 to 6 weeks
- ○ More than 6 weeks
- ○ Unsure

Approximately, what is the **most number of times <u>you</u>** visited stores to buy infant formula for your baby in a <u>24-hour period</u> during the **May 2022 infant formula shortage**?

[For this question include the same store more than once if you visited it more than once in a 24-hour period.]

- 1
- 2 to 3
- 4 to 6
- 7 to 10
- More than 10
- Unsure

Approximately, what is the **farthest distance** in **miles <u>you</u>** traveled to buy infant formula at one store in a <u>24-hour period</u> during the **May 2022 infant formula shortage**?

- 0 to 5
- 6 to 10
- 11 to 15
- 15 to 20
- 21 to 25
- 25 to 30
- More than 30
- Unsure

Did you use any infant formulas from "Operation Fly"?

[The U.S. government recently enacted "Operation Fly" to help families get access to safe imported infant formulas. [Click here to see the list of formulas provided by Operation Fly.]]

- Yes
- No
- Unsure
- Decline to answer

Questions about your pregnancy and when your baby was born

Did you ever participate in a **breastfeeding class** or **breastfeeding support group** (in-person or online) ***during*** your **pregnancy** (before your baby was born)?

[This includes paid or community groups such as La Leche League or WIC.]

- ○ Yes
- ○ No
- ○ Unsure
- ○ Decline to answer

On a scale of 0 to 10 how **helpful** do you think this breastfeeding class or support group was in **helping you achieve your breastfeeding goals**?

[For this question, move the marker to the number that best represents how helpful this breastfeeding class or support group was in helping you achieve your breastfeeding goals. 0 equals not helpful at all, 10 equals very helpful and 5 is somewhere in the middle. Select "Unsure" if you are unsure.]

Not helpful at all										Very helpful	Unsure
0	1	2	3	4	5	6	7	8	9	10	☐

On a scale of 0 to 10 how **helpful** do you think a **free lactation support class** taught by lactation consultants ***during*** **pregnancy** would **help mothers achieve their breastfeeding goals**?

[For this question, move the marker to the number that best represents how helpful you think this breastfeeding class or support group would be in helping mothers achieve their breastfeeding goals. 0 equals not helpful at all, 10 equals very helpful and 5 is somewhere in the middle. Select "Unsure" if you are unsure.]

Not helpful at all										Very helpful	Unsure
0	1	2	3	4	5	6	7	8	9	10	☐

Where did you deliver your baby?

- ○ Birth Center (within a hospital or free-standing)
- ○ Home
- ○ Hospital
- ○ Other setting (please describe) ______
- ○ Decline to answer

How was your baby born?

- ○ Cesarean section, emergency
- ○ Cesarean section, scheduled
- ○ Vaginally (including water births)
- ○ Decline to answer

Did your baby spend any time in the **neonatal intensive care unit (NICU)**?

- ○ Yes
- ○ No
- ○ Unsure
- ○ Decline to answer

Did **you and your baby** share **skin-to-skin** contact within <u>two hours</u> of your baby's birth?

[Skin-to-skin contact is when your baby is placed directly on your chest and their bare skin touches your bare skin.]

- ○ Yes
- ○ No
- ○ Unsure

- ○ Decline to answer

What was your baby **fed** within the first 72 hours of your baby' birth?

[check all that apply]

- ☐ Mother's own milk (your breast milk)
- ☐ Donor human milk
- ☐ Infant formula
- ☐ Other (please describe) ______

Did you receive **breastfeeding support** from a **lactation professional** within the first 72 hours of your baby's birth?

[A lactation professional is a lactation consultant, lactation counselor, nurse, or midwife.]

- ○ Yes
- ○ No
- ○ Unsure
- ○ Decline to answer

Did you **pay** (out-of-pocket) to receive **breastfeeding support** from a **lactation professional**?

[Paying out-of-pocket means that this service was not covered by your medical insurance.]

- ○ Yes
- ○ No
- ○ Unsure
- ○ Decline to answer

Why did you **not** receive **breastfeeding support** from a **lactation professional**?

[check all that apply]

- ☐ A lactation professional was not available at the times I needed
- ☐ I did not think I needed help from a lactation professional

- [ ] I was not offered the opportunity to meet with a lactation professional
- [ ] I was only able to meet with a lactation professional virtually and did not think it would be helpful to meet with them online
- [ ] Seeing a lactation professional was too expensive
- [ ] Seeing a lactation professional was not covered by my insurance
- [ ] Other ____________
- [ ] None of the above

Did you ever participate in a **breastfeeding class** or **breastfeeding support group** (in-person or online) ***after*** your baby was born?

[This includes paid or community groups such as La Leche League or WIC.]

- ○ Yes
- ○ No
- ○ Unsure
- ○ Decline to answer

On a scale of 0 to 10 how **helpful** do you think this breastfeeding class or support group was in **helping you achieve your breastfeeding goals**?

[For this question, move the marker to the number that best represents how helpful this breastfeeding class or support group was in helping you achieve your breastfeeding goals. 0 equals not helpful at all, 10 equals very helpful and 5 is somewhere in the middle. Select "Unsure" if you are unsure.]

Not helpful at all										Very helpful	Unsure
0	1	2	3	4	5	6	7	8	9	10	☐

On a scale of 0 to 10 how **helpful** do you think a **free lactation support class** taught by lactation consultants ***after* a baby is born** would **help mothers achieve their breastfeeding goals**?

[For this question, move the marker to the number that best represents how helpful you think this

breastfeeding class or support group would be in helping mothers achieve their breastfeeding goals. 0 equals not helpful at all, 10 equals very helpful and 5 is somewhere in the middle. Select "Unsure" if you are unsure.]

Not helpful at all										Very helpful	Unsure
0	1	2	3	4	5	6	7	8	9	10	☐

Lactation - Moms that breastfed baby only

Questions about breastfeeding your baby born between November 2021 to May 2022

Did you ever plan to **exclusively breastfeed** or exclusively feed expressed breast milk to your baby **without** the use of infant formula?

[Exclusive breastfeeding means feeding your baby only breast milk, not any other foods or liquids (including infant formula or water), except for medications or vitamin and mineral supplements.]

- ○ Yes
- ○ No
- ○ Unsure
- ○ Decline to answer

Did you **exclusively breastfeed** or exclusively feed expressed breast milk to your baby for as long as you had planned?

- ○ Yes
- ○ No
- ○ Unsure
- ○ Decline to answer

Is your baby currently breastfed or fed your expressed breast milk?

- ○ Yes

- No
- Unsure
- Decline to answer

How old was your baby when you **completely stopped** breastfeeding or feeding your expressed milk to your baby?

[Please answer in weeks OR months only. If your baby was younger than 1 week, please write "1" in the "weeks" box. If you have multiple babies (for example, twins or triplets, etc.) you may answer the same response for all of these babies.]

Weeks: ______

Months: ______

Did you breastfeed as long as you wanted to?

- Yes
- No
- Unsure
- Decline to answer

End of Questionnaire

Please share your thoughts about how you have dealt with the infant formula shortage crisis and the actions you feel should be taken by health authorities, food companies, and the government that could help you feed your baby during this crisis and prevent future crises.

Write "none" if you do not wish to leave a comment.

Please list an **email address** where we can send you the electronic $50 Target gift card if you are one of the first 100 respondents to complete it.

[Notifications to gift card recipients will be made in February 2023.]

[In order to receive a gift card, only one parent of one baby from the same household may complete this survey.]

Would you like to be contacted for future research opportunities from UC Davis?

- Yes
- No

Please write your email address so we can send you information

End of Questionnaire

Click the **next arrow** once you have completed the survey. Once you click the next arrow, you will not be able to go back to see your answers.

Powered by Qualtrics
